# Enrichment of Verrucomicrobia, Actinobacteria and Burkholderiales drives selection of bacterial community from soil by maize roots in a traditional *milpa* agroecosystem

**DOI:** 10.1371/journal.pone.0208852

**Published:** 2018-12-20

**Authors:** Eneas Aguirre-von-Wobeser, Jorge Rocha-Estrada, Lori R. Shapiro, Mayra de la Torre

**Affiliations:** 1 Cátedras CONACyT, Centro de Investigación y Desarrollo en Agrobiotecnología Alimentaria (Consortium between Centro de Investigación y Desarrollo, A.C. and Centro de Investigación y Asistencia en Tecnología y Diseño del Estado de Jalisco), Pachuca Ciudad del Conocimiento y la Cultura, San Agustín Tlaxiaca, Hidalgo, Mexico; 2 Department of Microbiology and Immunobiology, Harvard Medical School, Boston, Massachusetts, United States of America; 3 Centro de Investigación y Desarrollo en Agrobiotecnología Alimentaria (Consortium between Centro de Investigación y Desarrollo, A.C. and Centro de Investigación y Asistencia en Tecnología y Diseño del Estado de Jalisco), Pachuca Ciudad del Conocimiento y la Cultura, San Agustín Tlaxiaca, Hidalgo, Mexico; Free University of Bozen/Bolzano, ITALY

## Abstract

*Milpas* are rain-fed agroecosystems involving domesticated, semi-domesticated and tolerated plant species that combine maize with a large variety of other crop, tree or shrub species. *Milpas* are low input and low-tillage, yet highly productive agroecosystems, which have been maintained over millennia in indigenous communities in Mexico and other countries in Central America. Thus, *milpas* may retain ancient plant-microorganisms interactions, which could have been lost in modern high-tillage monocultures with large agrochemical input. In this work, we performed high-throughput 16S ribosomal DNA sequencing of soil adjacent to maize roots and bulk soil sampled at 30 cm from the base of the plants. We found that the bacterial communities of maize root soil had a lower alpha diversity, suggesting selection of microorganisms by maize-roots from the bulk-soil community. Beta diversity analysis confirmed that these environments harbor two distinct microbial communities; differences were driven by members of phyla Verrucomicrobia and Actinobacteria, as well as the order Burkholderiales (Betaproteobacteria), all of which had higher relative abundance in soil adjacent to the roots. Numerous studies have shown the influence of maize plants on bacterial communities found in soil attached tightly to the roots; here we further show that the influence of maize roots at *milpas* on bacterial communities is detectable even in plant-free soil collected nearby. We propose that members of Verrucomicrobia and other phyla found in the rhizosphere may establish beneficial plant-microbe interactions with maize roots in *milpa*s, and propose to address their cultivation for future studies on ecology and potential use.

## Introduction

There is increasing awareness of the importance of microbial communities for crop health and yield in agricultural systems [1–6]. Multiple studies have measured the diversity of plant-associated microbial communities both in agricultural systems and undisturbed environments [7, 8]. Some of these studies find significantly less microbial diversity in high input modern industrial monocultures [9], while others detect the dramatic changes in microbial communities that result from plant domestication [10] and natural land conversion to agriculture [11, 12].

In contrast, the study of microbial diversity in traditional agricultural systems has received considerably less attention, likely because the products from these systems are consumed locally, it is rather considered family farming and it is not part of the global commodity agriculture. In Mesoamerica—which encompasses present day central and southern Mexico, Belize, Guatemala, El Salvador, Honduras and Costa Rica—the traditional agroecosystems are called ‘*milpa’*, which is the Nahuatl word meaning ‘maize field’. *Milpas* have been cultivated for millennia and are low input and low tillage, yet highly productive agroecosystems. *Milpa* systems are highly diverse polycultures, but all have in common the cultivation of maize. The most common *milpa* crop co-culture is based around maize (*Zea mays* L.) as the main crop, and includes bean (*Phaseolus* spp.), which has the ability to fix nitrogen, and squash (*Cucurbita* spp.) for groundcover, water retention, and pollen and nectar source for natural enemies [13, 14]. *Milpa* systems throughout Mesoamerica often include many other locally cultivated crop species—notably chile, tomato, avocado, and agave—as well as dozens of domesticated edible herbs (*quelites*) and wild plants [15, 16], depending on local preferences and traditions.

*Milpa* farmers have carefully selected their own maize and other crops varieties for generations, resulting in a vast amount of heritage landrace diversity that is locally endemic and found nowhere else in the world [17, 18, 19, 20]. These individual landraces, and the polycultural cultivation system itself, were selected from the local biotic and abiotic environment by farmers to optimally utilize local ecosystem services. For example, polyculture crops flower at different times of day and at different times throughout the growing season, providing constant and varied nutritional sources for herbivore natural enemies and for pollinators. *Milpa*-farmers use neither synthetic pesticides nor fertilizers; crop plants must defend themselves against biotic pressures such as pathogens, herbivores, and against abiotic pressures like drought stress, and low nutrient levels. Thus, it is likely that plants at *milpas* rely on microbes as symbionts to endure these pressures, and that these agroecosystems differ from those found at modernized agricultural settings.

Here we studied the microbial communities found in the soil of *milpas*, which may establish unique plant-microbe interactions with biotechnological potential. We are certain *milpa* microbial communities must be studied *in situ—i*.*e*., within the ecological and human cultural context in which these agroecosystems have been cultivated for centuries—in order to accurately capture the endemic microbial function and diversity [21].

Most previous microbiome studies of maize rhizosphere use samples of soil tightly adhered to the roots, but we wanted to evaluate to what extent plants can shape the *milpa* soil communities close to the roots, so we sampled soil adjacent to the root system, as well as bulk soil to evaluate the effect of plant roots on microbial diversity. We used 16S ribosomal DNA sequencing, and assayed whether a local landrace maize variety is able to enrich distinct microbial communities from bulk soil. We found extensive changes in bacterial diversity and community structure in the presence of maize. Alpha biodiversity was lower in maize-root soil as compared to bulk soil, and there were changes in beta-diversity, indicating overall differences in the microbial communities. At the phylum level, we found that maize roots caused a decrease in the abundance of Gemmatimonadetes, and notably, an increase in Verrucomicrobia, a phylum with very few cultivable representatives, which has been proposed to establish favorable interactions with plant roots [12]. There were also many changes at the Sequence Variant (SV) level, with increased abundance of representatives from the phyla Verrucomicrobia and Actinobacteria, as well as the order Burkholderiales (Betaproteobacteria). Based on these results, our work shows the importance of focusing on the understudied Verrucomicrobia in the search for beneficial interactions that could be used for agrobiotechnology, and provides new evidence supporting unique diversity of root associated microbial communities in traditionally managed agroecosystems. Our results will help provide a foundation for future functional characterizations of agriculturally relevant microbial isolates and communities.

## Methods

### Characterization of the study area

We studied the *milpa* agroecosystem in the indigenous community of El Boxo, in the State of Hidalgo, Mexico (Coordinates for the data set: 20° 40' 37.9164"N, 99° 8' 39.4584"W). The study was carried out on private land, and the owner of the land gave verbal permission to conduct the study on the site. No specific permissions were required to collect samples from the studied milpa, as the land is private property, and the field studies did not involve endangered or protected species. We interviewed the *milpa’s* owners to learn about their agricultural practices and the plants and trees they seed associated to maize, as well as edible weeds allowed to grow in the *milpa*, and obtained verbally expressed informed consent of all participants for the publication of the interview. The native maize varieties cultivated were characterized using information from the website of the Mexican National Commission for Biodiversity (CONABIO).

### Soil sampling

On December 7, 2016, when the maize plants were mature and ready for harvest, we sampled soil using an auger (2 cm caliber). The plants selected for sampling belonged to the Conic landrace maize that produces white corn, and were located adjacent to each other, in the center of the parcel. Maize-root soil samples were collected directly adjacent to the maize plants bases, inserting the auger (rinsed and cleaned with ethanol) 15–20 cm, at an angle of approximately 45°, to collect a soil core. About 10 g of the root-containing segment, close to 10 cm depth, were collected using ethanol-cleaned knife and a spoon. Bulk soil control samples were taken at 30 cm from each sampled plant, avoiding any visible roots. Samples were immediately stored in liquid nitrogen, transported to the laboratory and kept at -70°C until DNA extraction. In contrast with most previous studies [22, 23, 24, 25], for the maize-roots samples, we removed visible maize roots, and extracted DNA from the surrounding soil. We also collected samples of soil for physicochemical analysis with a shovel in zip-lock bags and sent them to a specialized laboratory that uses standard methods for analysis of particle size (sand, clay and silt), pH, organic matter and nutrients.

### DNA extraction and 16S rRNA amplification

DNA was obtained from approximately 0.25 g of each sample of plant-free soil using a PowerSoil (Mo Bio Laboratories, Inc., USA) extraction kit, following the manufacturer’s instructions. Genomic DNA of each sample was quantified using Qubit technology, adjusted to 20 ng/μl and used as templates for PCR. Each sample was amplified in triplicate using primers F515 (5’-GTGCCAGCMGCCGCGGTAA-3’) and R806 (5’-GGACTACHVGGGTWTCTAAT-3’) specific for the V4 region of the 16S rRNA [26]. Oligonucleotides used for priming the PCR reactions also contained an adapter, primer pad and linker for Illumina sequencing; additionally, each reverse primer contained a unique 12-base GoLay barcode (S1 Table). Each PCR reaction contained 0.25 μl of Q5 High Fidelity polymerase (New Englan Biolabs), 0.5 μl of 10 mM dNTPs, 500 nM of each primer and 5 μl of 5X Q5 reaction buffer in a total volume of 25 μl. Reaction conditions were the following: 98°C for 30 s, then 35 cycles of 98°C for 10 s, 60°C for 30 s, and 72°C for 20 s; finally, 72°C for 2 min. PCR products were pooled, gel-purified, and their concentration was obtained using Qubit.

### Library preparation and Miseq sequencing

Pooled quantified library was processed for MiSeq sequencing by diluting to 4 nM and then denatured by mixing 1:1 with 0.2 N NaOH, for a final concentration of 2 nM of DNA and 0.1 N NaOH. Then, the library was mixed at a 1:1 ratio with 2 nM denatured PhiX Sequencing Control (Illumina). Three primers were used for sequencing: read 1 (5’-TATGGTAATTGTGTGCCAGCMGCCGCGGTAA-3’), read 2 (5’-AGTCAGTCAGCCGGACTACHVGGGTWTCTAAT-3’) and index (5’-ATTAGAWACCCBDGTAGTCCGG CTGACTGACT-3’). We used the MiSeq kit V2 for 500 cycles (Illumina) for sequencing, following the manufacturer’s instructions.

### Sequence processing and cleanup

The raw sequencing reads were pre-processed using QIIME 2 [27] following standard procedures. Barcoded reads were first demultiplexed to obtain 554,745 reads. The sample with least reads had 34,870 (6.37%; S2 Table), while the best covered sample had 104,479 (18.8%; S2 Table). To remove noise from the data, the first 13 and 28 nucleotides were removed from the forward and reverse reads, respectively, according to visual inspection of the quality at each position. This denoise-step was conducted using DADA2 [28], which also removed chimeras, contaminating sequences and combined the forward and reverse reads. After cleaning and preprocessing, the reads from all samples were aligned to the V4 region of a curated reference database of 16S sequences [29] to construct a table of SVs, equivalent to 100% Operational Taxonomic Units (OTUs). A total of 3849 SVs were found, with a length between 238 and 282 bases, and 93% being 249 bases long (S3 Table).

### Alpha diversity, beta diversity and community structure

Alpha and beta diversity analyses were conducted using the QIIME 2 plug-in q2-diversity. Some of these analyses, including phylogenetic diversity measures, required a phylogenetic tree. For that end, the sequences were aligned using MAFFT [30]. Highly variable regions were masked from the alignment, and a phylogenetic tree was constructed using FastTree [31]. Finally, the tree was rooted at the midpoint of the longest distance between tips, as described in the QIIME 2 documentation.

As measures of alpha biodiversity, the number of observed SVs, as well as Shannon Index, Faith’s Phylogenetic Diversity and Evenness were calculated. Since these indexes depend on the sampling depth (total number of reads obtained in each sample), for their calculation, the reads were randomly re-sampled to the lowest sampling depth of all the samples (34,870). To test for significant differences of alpha biodiversity indexes between maize-root samples and controls, non-parametric Kruskal-Wallis tests were performed. To further compare the alpha biodiversity indexes, they were calculated at increasing sampling depths, from 1,000 to 10,000, and plotted as rarefaction curves.

For beta diversity, the Bray-Curtis dissimilarity, Jaccard Index, as well as Weighted and Unweighted UNIFRAC [32] distances were calculated between all samples. In order to identify groupings of samples via ordination, the dimensionality of these beta-diversity measures was reduced by Principal Coordinates Analysis (PCoA). For the statistical analysis of the beta-diversity measures, we assessed the statistical significance of the differences between the centroids of the two sample groups (maize-root samples vs controls). The centroids are the means of all the points in all of the principal coordinate axes for a given beta-diversity measure, and their difference was tested using PERMANOVA [33] with 999 permutations, in QIIME 2.

To identify the taxonomic affiliation of the SVs, from phylum to genus, they were aligned to the reference database SILVA [29] in QIIME 2. The statistical significance of differences in the abundances of phyla in maize-root samples and controls was assessed using Wilcoxon tests at the 95% significance level. Differences in the abundance of SVs were also tested with Wilcoxon tests. Only SVs that were present in 5 or more of the 10 samples were considered for these analyses. These SV analyses implied a large number of tests (588). However, due to the discontinuous distribution of p-values resulting from the Wilcoxon tests, it was not possible to calculate False Discovery Rates (FDR) [34]. Thus, we reported a list of SVs with p-values of less than 0.05. All those SVs showed marked differences in the median values between groups. Nevertheless, we note that some false positives may be included.

### Phylogenetic tree of SVs differing between sample-groups

To evaluate the phylogenetic relationships of the SVs that differed between maize-root samples and controls, a tree was constructed for those sequences. Using BLAST [35], closely related sequences were identified for use as references. All included sequences were aligned with MUSCLE [36], and the tree was constructed with Maximum Likelihood estimation using RAxML [37] with default parameters. The tree was plotted using the package ggtree in R [38].

### Sequence availability

The sequence raw data are available at the Short Read Archive (NCBI), with accession number SRP140584, as BioSamples SAMN08939694, SAMN08939695, SAMN08939696, SAMN08939697, SAMN08939698, SAMN08939699, SAMN08939700, SAMN08939701, SAMN08939702 and SAMN08939703, one for each sample, within BioProject PRJNA450448.

## Results

### Characterization of the *milpa*

For this study, a traditional *milpa* was selected at El Boxo community in the state of Hidalgo, Mexico (S1 Fig). The *milpa* is located at 20° 40' 37.9164" latitude north and 99° 8' 39.4584" longitude west, 2350 meters above sea level. The *milpa* is managed by its Otomi-speaking owner, Don Pedro Rivera, with minimum tilling between seasons (S1 Interview). The main crop was maize; four varieties of native maize, belonging to two landraces, were cultivated. The race “Elotes Conicos” was represented by one white and one red variety, while the race “Conico” had a blue and a variegated variety (S2 Fig). Alongside maize, the other cultivated plants included beans (*Phaseolus* spp.), fava beans (*Vicia faba)*, squash (*Cucurbita* sp.), chili (*Capsicum* sp.) and tomato (*Solanum lycopersicum*). Several fruit-trees were present at the *milpa*, including pear, plum, bitter-berry, tejocote (*Crataegus mexicana)*, apricot and apple. The wild plants included several edible weed species (generically termed “quelites”). Fourteen plants, 5 fungus and 5 animal products were found at the *milpa*.

Maize is cultivated from May through December, and sheep pasture during the rest of the year. The farmers seed directly, leave the corn stubble in the field and seldom use agrochemicals; sheep residues work as fertilizers. Indeed pest and phytopathogens are not a problem. Unlike swidden agroforestry systems [39], this *milpa* is permanently devoted to agriculture, and therefore not reliant on forest-burning practices. At the time of sampling, the maize plants in the *milpa* were fully grown and ready to harvest (Maize growth stage R6). All the plants selected for sampling were “Elote-Conico” landrace and produced white corn.

A soil sample was analyzed from the *milpa* agroecosystem of El Boxo in order to classify the soil type and its dominant physical and chemical characteristics. According to the proportions of sand, clay and silt (S4 Table), the corresponding soil texture is clay loam, with neutral pH, mild organic matter and rich in nutrients. The soil has an excess of phosphate and nitrate, as well as adequate to high concentrations of sulphate, potassium, magnesium, copper and zinc (S4 Table).

### Soil adjacent to *milpa* maize-roots has decreased bacterial diversity

**Fig 1:**
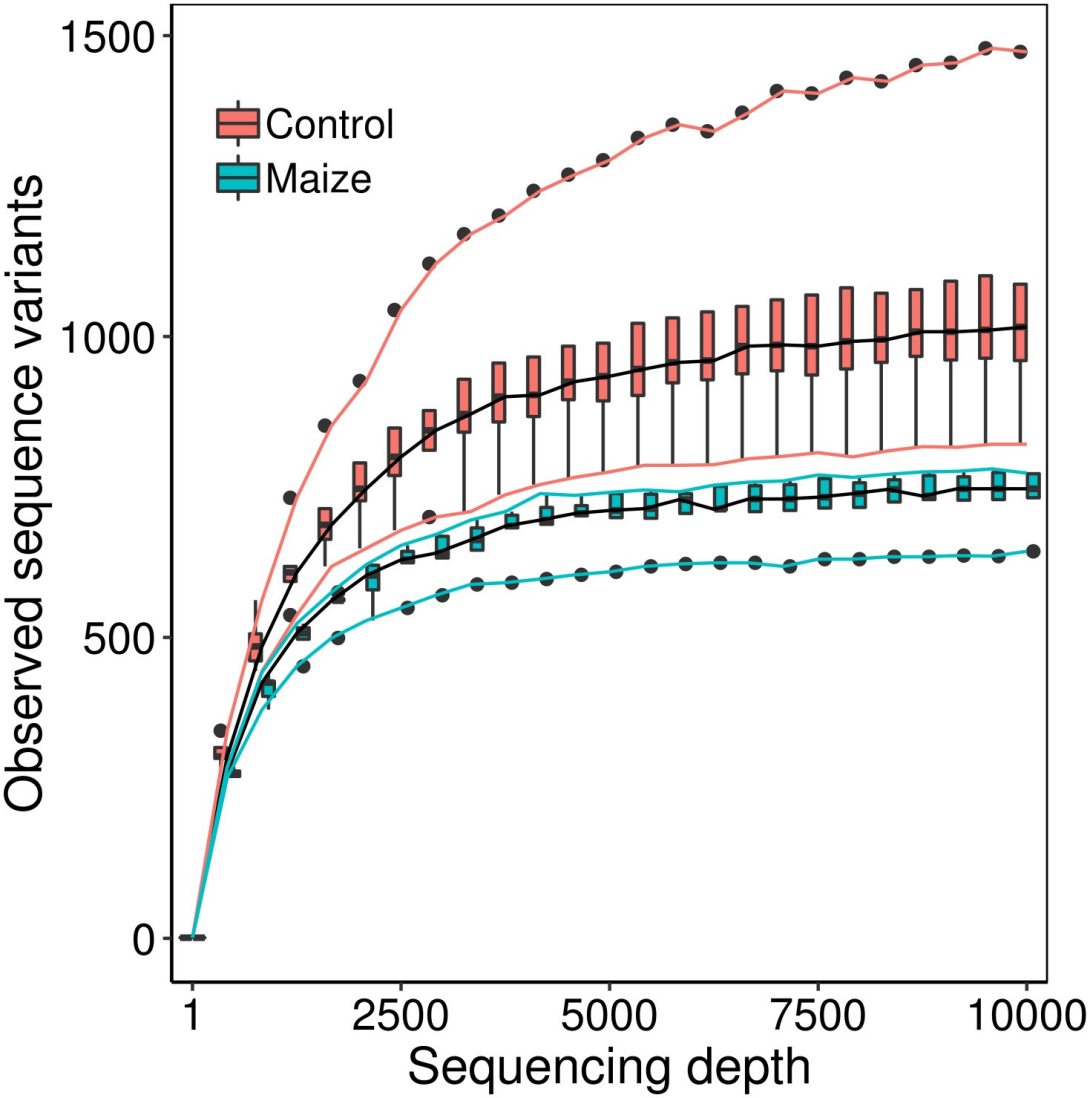
Rarefaction curve for the Sequence Variant number (richness). At each sampling depth 10 random samples were taken, and the distribution of Sequence Variant numbers for all 5 samples per group are shown as boxplots (50 values per box plot). Maize-root soil samples are shown in blue, and bulk soil controls in red. Continuous lines show the most extreme values at each sampling depth.

To analyze the effects of the presence of maize plants on the soil biodiversity at small-scale, we calculated different diversity indexes on the detected SVs. Since these indexes increase as more observations are collected, we used rarefaction curves, where data were randomly sub-sampled to equal increasing numbers, from 1,000 to 10,000 reads. The curves flattened at relatively low sequencing depths (Fig 1), indicating that the sequencing effort was adequate to capture most of the diversity in the samples, and the sequencing run was representative of the SVs present in the soil.

We found that most microbial biodiversity measures were lower in the presence of maize plants as compared to bulk soil. The SV number was higher in bulk soil compared to maize-root soil at all rarefaction sampling depths (Fig 1), indicating that the presence of maize roots reduced the SV richness of the soil bacterial community. Alpha diversity measured via the Shannon index and Faith’s PD were also lower in maize-roots soil samples at all rarefactions (S3 Fig). We contrasted the differences in the diversity indexes between maize-roots soil samples and bulk soil at a sampling depth corresponding to the sample with the lowest number of sequencing reads (34,870) with Kuskal-Wallis tests. SV number, Shannon Diversity and Faith’s PD were significantly lower in maize-roots soil samples than in bulk soil (H = 6.82, p-value = 0.009023 for all three tests). There were no differences in the evenness index (which measures how equal the abundances of the different SVs are) between the two sampling groups (H = 0.88, p = 0.3472).

### *Milpa* maize-roots select specific taxonomic groups from bulk-soil bacterial community

**Fig 2:**
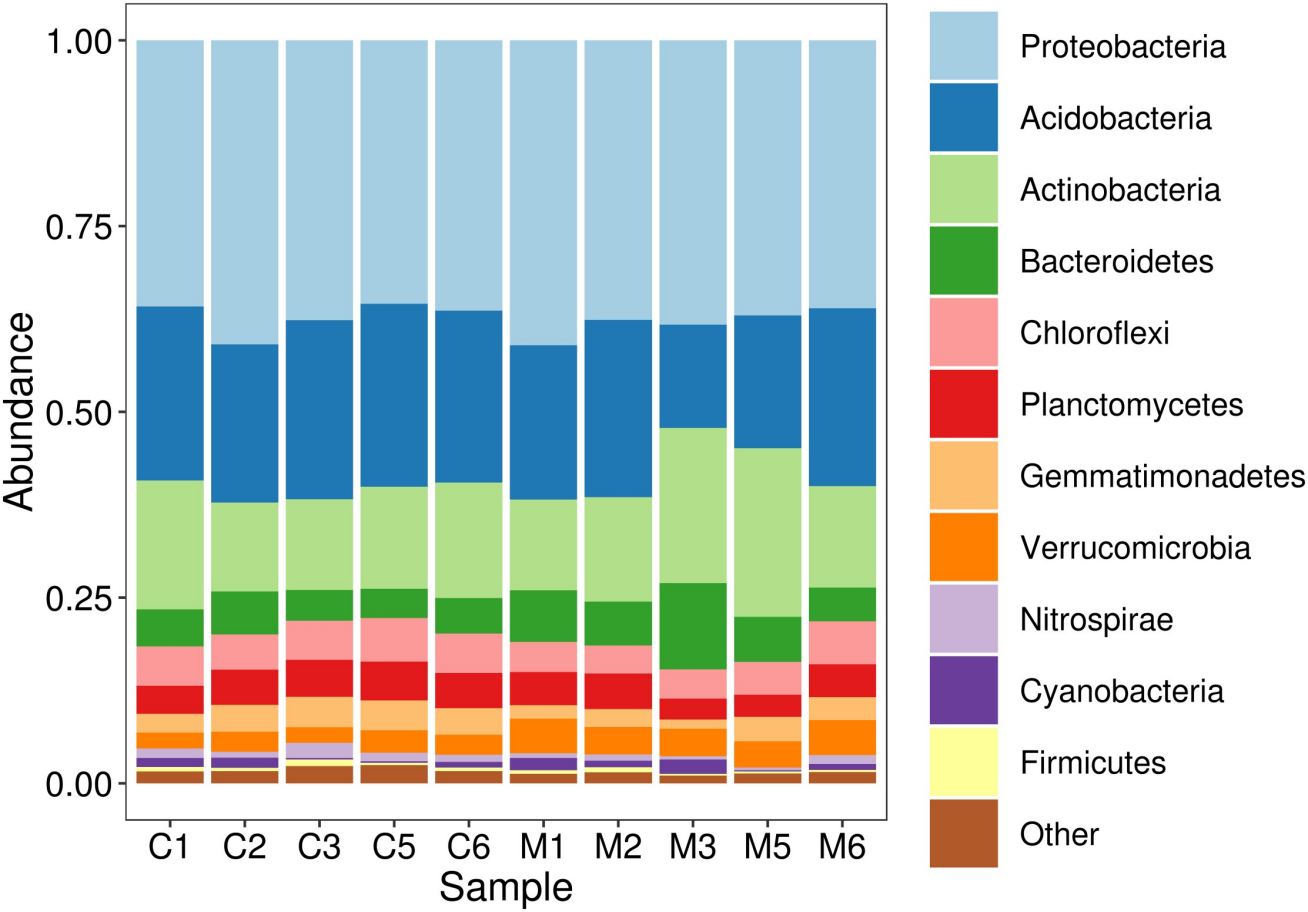
Histogram of the abundance of phyla of soil samples from the *milpa* at El Boxo. Soil samples named with an initial "M" were taken at maize-plants roots, and samples named with an initial "C" are bulk-soil controls, taken at 30 cm from the plants. Phyla with relative abundance higher than 2% are shown.

**Fig 3:**
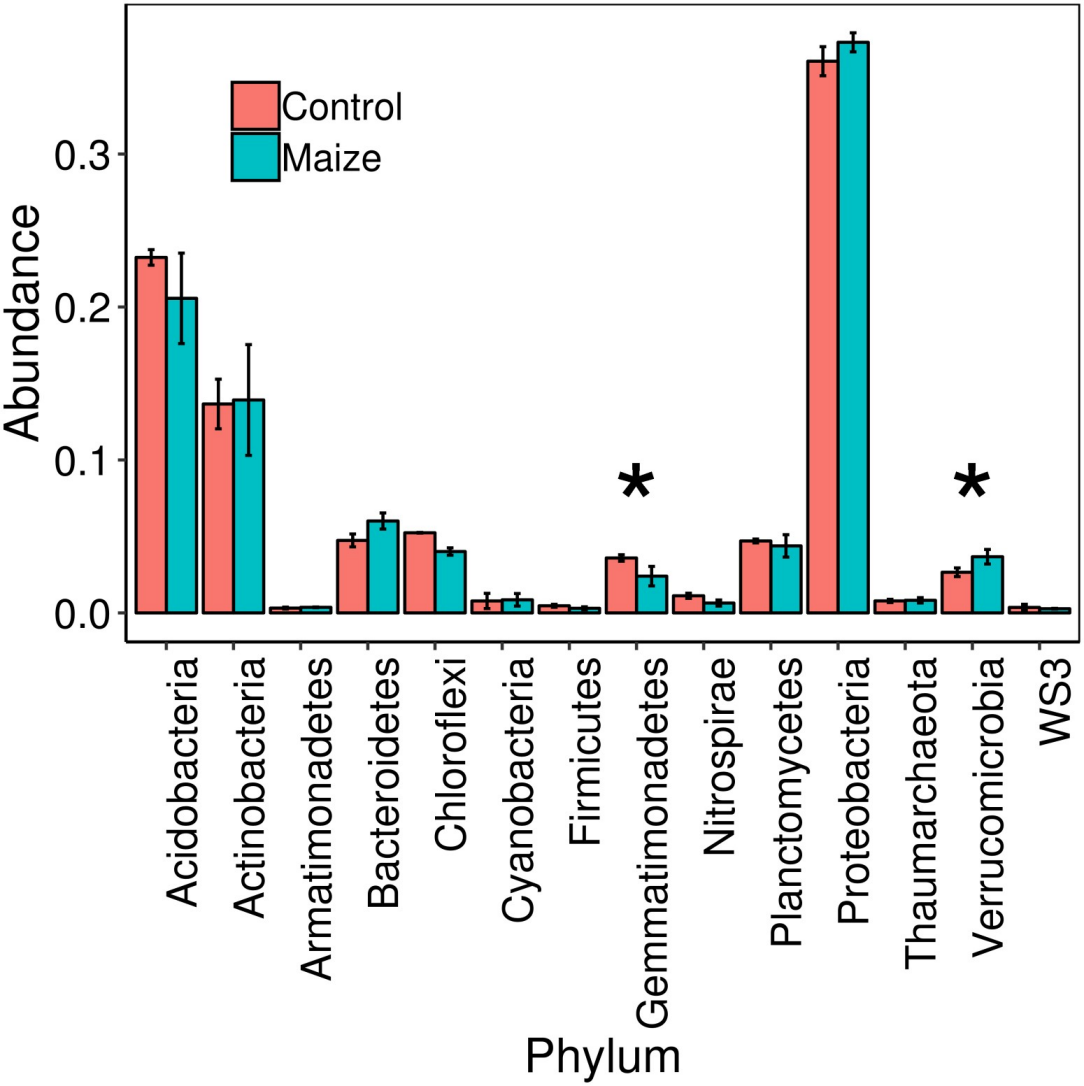
Abundance of phyla of soil samples from the *milpa* at El Boxo. Values are medians, and error bars are interquartile ranges. Bars in blue are soil samples taken at maize-plants roots and red bars are bulk soil controls. Asterisks indicate statistically significant differences (Wilcoxon, p < 0.05).

For assigning taxonomy, the obtained SVs were aligned to a reference database and identified from phylum, down to the genus level. Overall, in both maize-roots and bulk soil samples, the most abundant phyla identified (Fig 2) were Proteobacteria (37.6%), Acidobacteria (21.7%), Actinobacteria (15.4%), Bacteroidetes (5.9%), Chloroflexi (4.8%), Planctomycetes (4.3%), Verrucomicrobia (3.3%) and Gemmatimonadetes (2.9%). The abundances of most phyla did not differ between bulk-soil and maize-root soil samples (Fig 3; S5 Table). However, the phylum Gemmatimonadetes had significantly lower abundance in maize-root soil samples (Wilcoxon, p < 0.05), suggesting that this phylum may be specifically repressed by the presence of the plants. Conversely, the phylum Verrucomicrobia was enriched in maize-roots, as an increased abundance was found in maize-roots soil samples, compared to bulk soil (Wilcoxon, p < 0.05).

**Fig 4:**
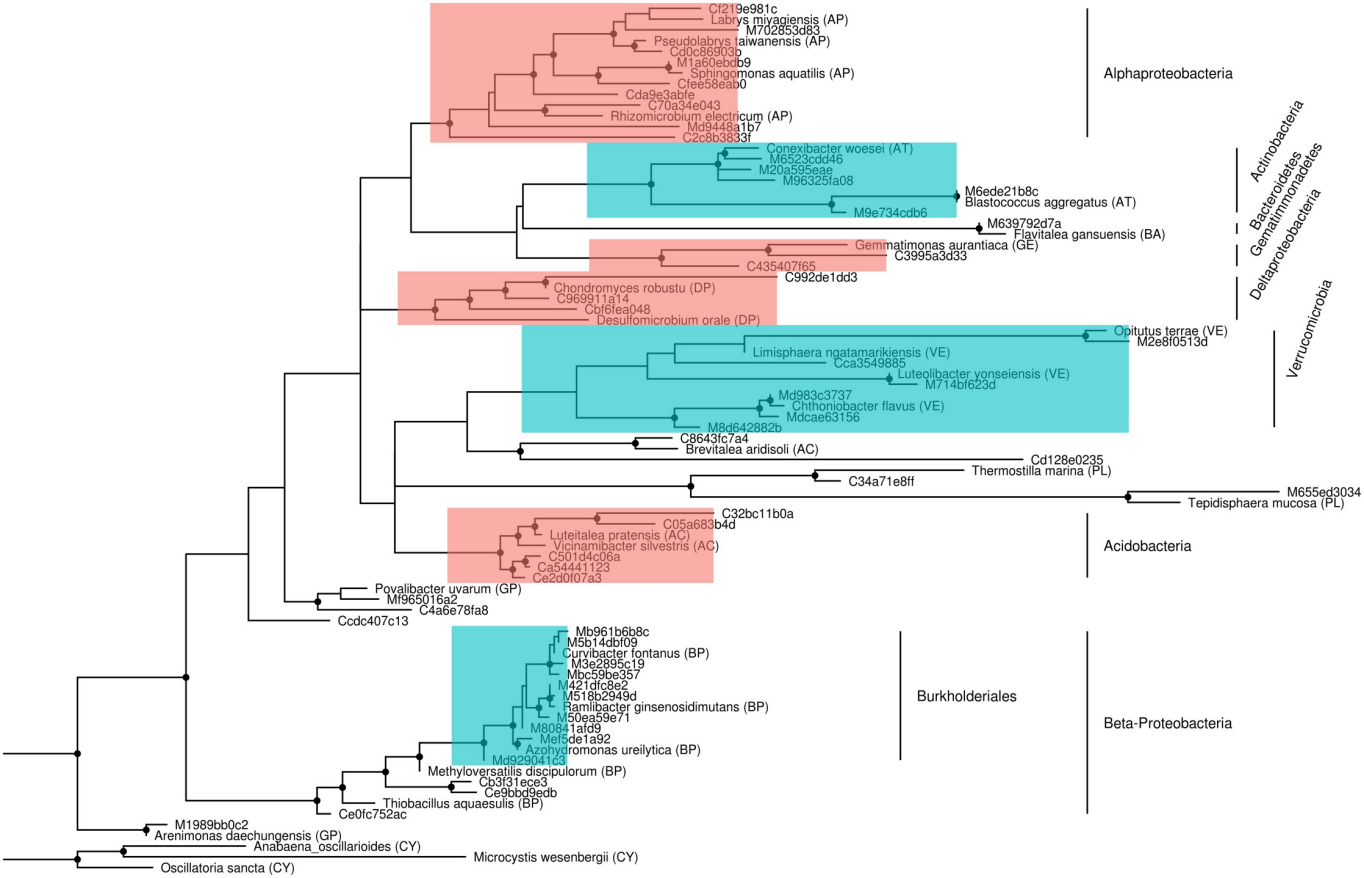
Phylogenetic tree of sequence variants with large differences of abundance between soil sample-groups from El Boxo *milpa*. Sequence variants starting with M are significantly more abundant in maize-root soil samples, and sequence variants starting with C are more abundant in bulk soil controls. Three cyanobacteria species were used as an outgroup. Color highlights indicate branches with a large majority of sequence variants with more abundance in maize (blue) and bulk soil (red).

Overall, the abundance of different SVs was very similar between the bulk soil and maize-root soil. The median abundances of in soil near maize-roots and bulk soil clearly followed a linear x = y relationship (S4 Fig). However, there was considerable dispersion around this trend, so we performed statistical tests to compare the relative abundance of each SV in both groups of samples. Wilcoxon tests resulted in 54 SVs with low p-values (p < 0.05; S6 Table), suggesting these 54 SVs had either higher or lower abundance in one of the sample types. Furthermore, 21 SVs found in bulk soil were not detected in the maize-roots samples, and only one SV found in the maize roots soil was absent from all bulk soil samples. The SVs with differential abundance belonged to taxonomically diverse phylogenetic groups (Fig 4), indicating that maize plants shape the composition of microbiota in soil close to their roots. SVs enriched in maize-roots soil samples included representatives from the phyla Verrucomicrobia and Actinobacteria, as well as the order Burkholderiales (Betaproteobacteria). Bacteria with lower abundances at maize-root soil samples compared to bulk soil, included SVs from the phyla Gemmatimonadetes and Acidobacteria, and the classes Deltaproteobacteria and Alphaproteobacteria (mostly the order Rhizobiales).

### *Milpa* maize-root shapes overall community structure of soil

**Fig 5:**
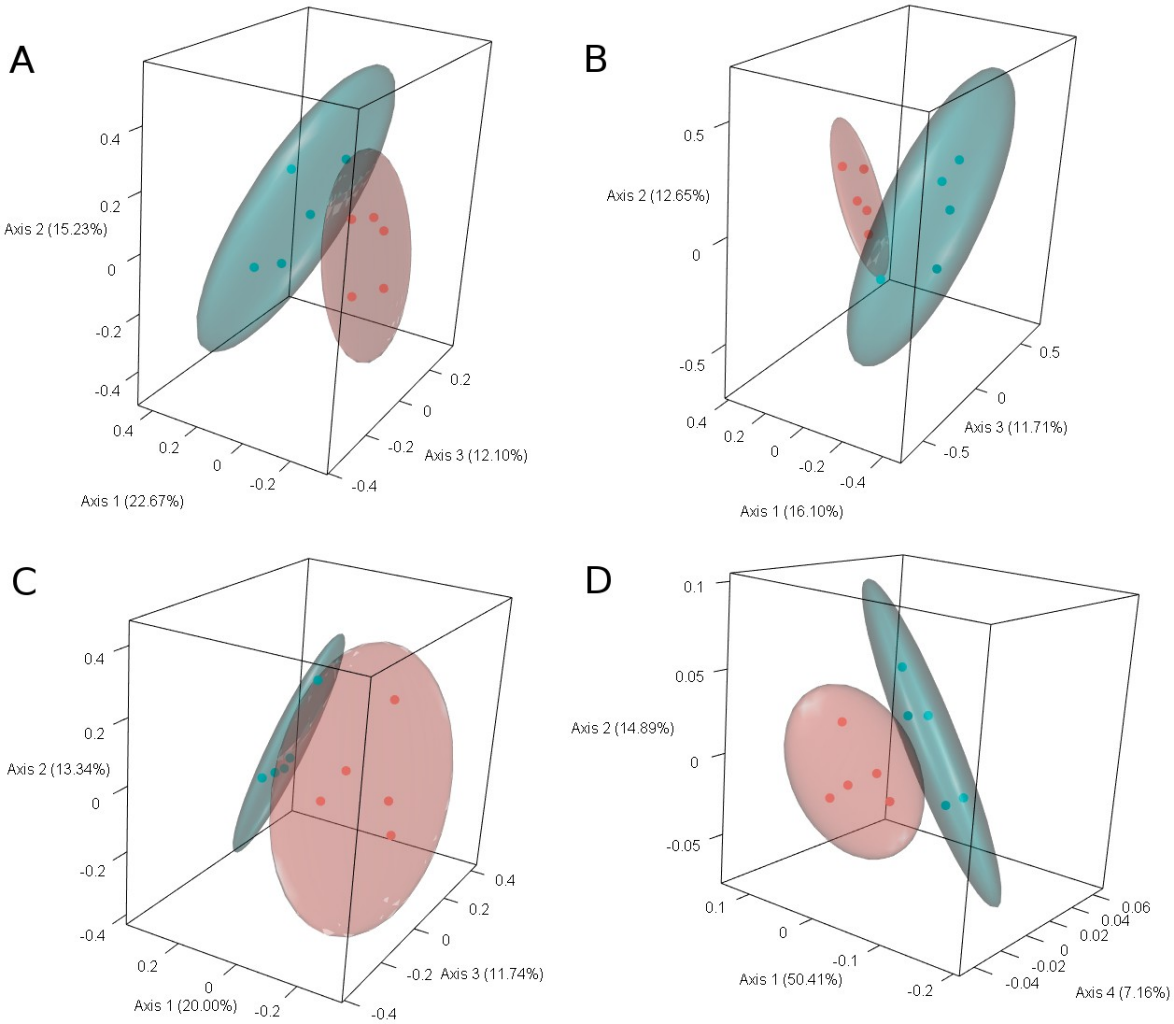
Beta diversity, expressed as Principal Coordinate Analysis of ecological distances between maize-roots soil samples (blue) and bulk-soil controls (red) taken at 30 cm from the plants. Ellipsoids represent 95% confidence intervals. A) Bray-Curtis dissimilarity, B) Jaccard index, C) Unweighted UniFrac and D) Weighted UniFrac.

We performed beta diversity analyses to determine whether the observed differences in presence and abundance of individual SVs between maize-roots soil and bulk soil samples had overall effects in the community structure. For this purpose, we used different ecological measures of relatedness, and summarized the results with Principal Coordinates Analysis (PCoA). With Bray-Curtis dissimilarities, samples from the two groups were clearly separated (Fig 5A), indicating quantitative differences between the bacterial community structures of maize-roots soil and bulk soil. This separation of the sampling groups was significant, as determined by Permutational Multivariate Analysis of Variance (PERMANOVA; p = 0.006; Table 1). Jaccard similarity and Unweighted UniFrac also clearly separated the maize roots and bulk soil samples in the PCoA ordination (Fig 5B and 5C; PERMANOVA test, p = 0.014 and 0.006, respectively). The separation of groups with weighted UniFrac was less marked (Fig 5D), and could be better observed using the 4^th^ PCoA axis instead of the 3^rd^; nevertheless, the separation of the sample-groups was also significant (PERMANOVA test, p = 0.024). Indeed, all of these standard measurements of ecological relatedness were able to differentiate the microbial communities from maize-roots soil *vs*. bulk soil.

**Table 1:** PERMANOVA test for group centroids.

	Df	SumsOfSqs	MeanSqs	F.Model	R2	Pr(>F)
Sample	1	0.11984	0.119842	3.4296	0.27592	0.005
Residuals	9	0.31449	0.034943	0.72408		
Total	10	0.43433	1.00000			

Note: Bray-Curtis distances were used. The analysis compared microbial communities from soil samples taken from maize roots with bulk-soil controls, taken at a distance of 30 cm from the plants.

## Discussion

The structure and function of soil bacterial biodiversity in traditional Mesoamerican *milpa* agricultural systems remains almost completely unknown; this is also true for overall ecological aspects of *milpas*, which remain largely understudied. We studied a *milpa* at El Boxo, Mexico, which was selected because of its high native crop agrobiodiversity and traditional, low-input management. Although several introduced species such as apple and pear trees were present at this *milpa*, the crop diversity and crop landraces are representative of the ancestral plants cultivated in this area for hundreds of years using the same farming system, *i*.*e*. conservation agriculture [14]. It is possible—even likely—that these systems have only endured for thousands of years in the absence of synthetic inputs because of the ecosystem services provided by local plant associated microbes.

We collected soil samples from a traditional polyculture *milpa* agroecosystem in Hidalgo, Mexico, to compare microbial diversity of bulk soil and soil in proximity to maize-roots. We found that maize-root soil samples had significantly lower alpha diversity and community richness as compared to the bulk soil sampled from a distance of only 30 cm from the roots sample (Fig 1). This significantly lower microbial diversity in the presence of the roots suggests that maize plants can exclude and/or select specific bacteria from their immediate surroundings; the same idea was proposed for commercial maize lines in modernized agricultural systems [40]. To the best of our knowledge, our study is the first one designed to determine the effects of maize plants-roots on the microbial communities of surrounding soil, compared to adjacent bulk soil. This contrasts with other high-throughput 16S sequencing studies, which analyzed maize roots with adhered soil particles [24, 22, 25], soil tightly attached to the roots [23], works that did not include bulk soil samples [41, 24] or only analyzed bulk soil on maize fields [42, 43]. In agreement with our results, soil tightly attached to the roots had a lower alpha diversity than bulk soil [22, 23]. In contrast, Rebollar et al. [44] did not find differences in alpha diversity between soil samples taken at 5 and 20 cm from maize plants in a *milpa* agroecosystem; perhaps at 5 cm the maize roots no longer influence the microbial community. Bakker et al. [45] analyzed soil attached to the roots and contrasted it with loose soil around them, but did not include a bulk soil sample separated from the roots. Our sampling method allowed us to detect a wider influence of maize-root microbiota selection, including the soil adjacent to the roots, but not attached to them.

The physicochemical characteristics of soil can strongly influence microbiota directly, or have effects on plant exudates [40, 45]. The soil of El Boxo-*milpa*, consists of clay loam, with neutral pH, mild organic matter content and rich in nutrients, and was dominated by the phyla Proteobacteria, Acidobacteria and Actinobacteria. These are also the common dominant phyla in other soil ecosystems, including tropical and temperate forest, pasture, arid woodland, alpine meadow and agricultural systems, as well as other Mexican *milpa* agroecosystems [7, 44, 46, 24, 23]. However, slight differences can be identified among studies. In a mesocosm experiment using different soil-types, the most abundant bacteria in clay soils were Proteobacteria, followed by Actinobacteria and Bacteroidetes [45]. El Boxo soil was a clay loam, but Acidobacteria was the third most abundant phylum instead of Bacteroidetes. Soil pH also has large effects on microbial communities [8, 47] and the most affected phyla are Acidobacteria, Actinobacteria and Bacteroidetes [47]. Neutral pH in soils favors Actinobacteria and Bacteroidetes [47], while Acidobacteria thrive on more acidic soils; nevertheless, we found 20% of Acidobacteria in the neutral soil of El Boxo. On the other hand, the percentage of Bacteroidetes in El Boxo is low compared to other neutral pH soils [47]. We can suggest that, although the most abundant phyla found in El Boxo *milpa* microbiome are in accord to other studies performed in soil with similar physicochemical characteristics, there are a few important variations that are unique to the microbial communities of our samples, that may be attributable to the particular biotic and abiotic conditions, including the low modernization in this agroecosystem.

In this work, we found that the presence of maize plants *in milpas* had an effect on the abundance of certain taxa as compared to bulk soil (Figs 3 and 4). Similarly, previous studies on root microbiomes of industrial inbred maize cultivars also found differences in major taxonomic groups (phyla and orders) as compared to bulk soil [22, 23]. Although these works are not directly comparable to our study, since they focused on soil tightly adhered to the roots, some similarities could be observed. For instance, Peiffer et al. [22] found that the presence of plat roots caused an increase in abundance of bacteria from order Burkholderiales, and increased abundance of phylum Acidobacteria, which is consistent with our results. Likewise, in study using a commercial maize cultivar, phylum Actinobacteria and order Burkholderiales were more abundant at the roots, while Gemmatimonadetes was less abundant [23], as observed in our study. However, results form those studies also had differences in respect to our results. For instance, an overall increase of Proteobacteria was observed in both works, which was interpreted as a result of the higher availability of labile organic carbon in the rhizosphere, which would benefit these copiotrophic bacteria [22, 23]. This suggested advantage of Proteobacteria at the rhizosphere could be limited to the immediate surrounding of roots, and only be observed in tightly adhered soil. Another difference was that one study found a decrease in abundance of Verrucomicrobia at the roots [22], while in the other this phylum was not detected as an abundant group [23]. Finally, Li et al. [23] found plant roots caused a decrease in abundance of phyla Chloroflexi, Firmicutes and Nitrospira, but we did not find such effect at El Boxo *milpa*. Overall, the differences observed could be related to the agricultural setting (industrialized vs. agroecosystem), maize variety (commercial hybrid vs. traditional landrace), sampling strategy (tightly attached soil vs. soil surrounding roots), among other differences. It is possible that the local biotic/abiotic environment and/or the genotype of the plant can affect the mechanism and outcome of maize roots microbial recruitment.

Even though the abundance of most phyla did not differ between bulk soil and soil from maize-roots, there were differences in their composition that were only observed at the SV level. We detected several SVs with significantly different abundances between maize-roots and bulk soil (Fig 4; S6 Table). This trend (general stability at the phylum level and differences at fine taxonomic levels) is common when comparing soil samples, for example, soils collected at distances of kilometers [48]. However, finding community differences of samples collected within 30 cm is surprising, as high spatial dispersion of bacterial cells would be expected at this scale. This is particularly true for agricultural fields, where soil is frequently disturbed during planting, human activity during the growing season, and harvest. The differences in SV abundances between maize roots soil and bulk soil were also reflected in the beta diversity patterns, suggesting maize roots have a robust effect on bacterial abundances at the community level. The heterogeneity of bacterial communities in our samples may be attributable to the less intrusive management in low till *milpa* systems and a strong effect of exclusion of certain SVs by the plant roots.

Only a small fraction of the total bacterial diversity can be grown in laboratory conditions; metagenomics can provide some information independent of our ability to culture these organisms, but it is hard to establish causative correlations from metagenome sequencing. However, we can infer the biological relevance of unculturable bacteria from bioinformatics data analysis combined with precise sampling methods. At the phylum level, we observed a significant reduction in the abundance of Gemmatimonadetes in the soil surrounding maize-roots. Gemmatimonadetes is a recently described phylum [49] with few cultured representatives, which is persistent in soil [50]. Gemmatimonadetes tend to be favored by low moisture [50], and include a strain with a functional photosystem II, but lacking C fixation genes [51]. The type strain of this phylum accumulates polyphosphate [49] and could reduce the phosphate availability for the plant. Thus, experiments are needed to elucidate the interactions of Gemmatimonadetes with *milpa-*crops to test whether the exclusion of members of this phylum from soil around maize-roots could be explained by competition for phosphate.

Verrucomicrobia is another phylum with few cultivable representatives. Interestingly, Verrucomicrobia was the only phylum, as a whole, with significantly increased abundance at the soil adjacent to maize roots, compared to bulk soil (Fig 3). Although little is known about the physiology of these bacteria, some representatives associated to rice plants have been shown to incorporate plant-derived carbon for growth [52]. For instance, we found that the SVs corresponding to *Chthoniobacter flavus* and *Opitutus terrae* were both significantly enriched around maize plant roots (Fig 4); these bacteria contain genes for the degradation of plant saccharides [53, 54]. Another study showed a positive correlation between the abundance of Verrucomicrobia and the presence of genes for degradation of complex carbohydrates in North American prairies [12]. Also, Nunes da Rocha et al. [55] showed that two Verrucomicrobia strains could grow on leek root exudates as the sole carbon source. Altogether, these findings suggest that Verrucomicrobial bacterial cells growing on or near the root surface may depend on carbon sources coming from the plant. Finally, Navarrete et al. [56] found a negative trend in Verrocomicrobia abundance with increasing soil fertility in sugarcane roots. This increased abundance of Verrucomicrobia in soils with lower fertility, defined as lower contents of nutrients like P and K, suggest they behave as k-strategists, with low growth-rates and high affinity for nutrient substrates [56]. Thus, Verrucomicrobia can be described as oligotrophs, and their abundance decreases when nitrogen is in excess [11, 12]. It has also been shown that different maize inbreds select for particular Verrucomicrobia [25]. The enrichment of Verrucomicrobia observed in our study in soil adjacent to the roots reflects selection of these particular bacteria from the microbial pool present at the *milpa* soil. Since the *milpa* in el Boxo has been cultivated for centuries, and every year the farmer has seeded together different maize varieties and races for inbreeding to obtain new characteristics, associated Verrucomicrobia strains may have co-evolved with maize for hundreds of years.

The changes in the abundance of certain bacterial groups in the presence of maize roots could guide the search for beneficial bacteria from traditional *milpas*. Verrucomicrobia, for example, are very abundant in soils, but only few species have been successfully cultivated *in vitro* [57]. It may be worthwhile to target this Phylum in future functional studies. Actinobacteria is one group of bacteria that is already being investigated for potential beneficial uses in agriculture, and a large variety of candidate strains with beneficial activities have been identified [58]. Actinobacteria are well known for their diverse secondary metabolism, with many ecological functions and potential uses [59]. Similarly, bacteria belonging to the Burkholderiales have been studied for their application in agrobiotechnology, for instance in pathogen suppression [60] or their potential to fix nitrogen in association with grasses, including agriculturally relevant staple grass crops [61, 62]. The enrichment of Actinobacteria and Burkholderiales in soil samples collected at the roots in our study supports the general view that maize plants might select for beneficial bacteria in this environment.

The study of microbial communities in traditional *milpa* agroecosystems is a first step towards harnessing these beneficial microorganisms for future biotechnological applications. Our results highlight the importance of focusing on bacterial groups recalcitrant to cultivation, like Verrucomicrobia. These bacteria may present useful interactions with plants, which could have been lost in modern high-tillage monocultures with large agrochemical inputs. We propose our results should guide future cultivation efforts, in search of bacterial strains or consortia that could be used to improve sustainable maize agriculture.

## Supporting information

S1 Table Primers used for sequencing library preparation. (PDF)

S2 Table Sequences per sample. (PDF)

S3 Table Length distribution of the clean, pre-pocessed, quimera-free DNA sequence variants. (PDF)

S4 Table Soil analysis parameters. (PDF)

S5 Table Wilcoxon tests for the differences in abundances of phyla (Only the 14 most abundant phyla were tested. (PDF)

S6 Table Wilcoxon tests for differences of sequence variants (SV) between maize-root samples and controls. (PDF)

S1 Fig Study area. Image of the *milpa*.
(JPG)

S2 Fig Maize varieties cultivated at the studied milpa. From left to rigth, Conico (white), Elotes Conicos (red), Elotes Conicos (black), Conico (variegated).
(TIFF)

S3 Fig Rarefaction curves. Rarefaction curve for A) Shannon diversity index and B) Faith’s phylogenetic diversity index. At each sampling depth 10 random samples were taken, and the indexes for all 5 samples per group are shown as boxplots (50 values per box plot). Maize-root soil samples are shown in blue, and controls in red. Continuous lines show the most extreme values at each sampling depth.
(TIFF)

S4 Fig Median abundances of all SVs in maize-root samples vs controls. (TIFF)

S1 Interview Interview with farmer Pedro Rivera. (PDF)
