# Supplementary material for: Enrichment of Verrucomicrobia, Actinobacteria and Burkholderiales drives selection of bacterial community from soil by maize roots in a traditional milpa agroecosystem
Source: PLoS One. 2018 Dec 20;13(12):e0208852. doi: 10.1371/journal.pone.0208852 (PMC6301694; doi:10.1371/journal.pone.0208852)
Supplement: S1 Table — (PDF) [file pone.0208852.s001.pdf]

S1 Table. Primers used for sequencing library preparation

Forward primers						
Sample		5' Illumina adapter		Forward primer pad	Forward primer linker	Forward primer Fwd515
All samples		AATGATACGGCGACCACCGAGATCTACAC		TATGGTAATT	GT	GTGCCAGCMGCCGCGGTAA
Reverse primers						
Sample	Primer number	Reverse complement of 3' Illumina adapter	GoLay Barcode	Reverse primer pad	Reverse primer linker	Reverse primer Rev806
Boxo1/85	806rcbc85	CAAGCAGAAGACGGCATACGAGAT	CAGCTAGAACGC	AGTCAGTCAG	CC	GGACTACHVGGGTWTCTAAT
Boxo1C.13	806rcbc13	CAAGCAGAAGACGGCATACGAGAT	CCTCGTTCGACT	AGTCAGTCAG	CC	GGACTACHVGGGTWTCTAAT
Boxo2.73	806rcbc73	CAAGCAGAAGACGGCATACGAGAT	GACGGAACCCAT	AGTCAGTCAG	CC	GGACTACHVGGGTWTCTAAT
Boxo2C.1	806rcbc1	CAAGCAGAAGACGGCATACGAGAT	AATCAGTCTCGT	AGTCAGTCAG	CC	GGACTACHVGGGTWTCTAAT
Boxo3.61	806rcbc61	CAAGCAGAAGACGGCATACGAGAT	ATGAGACTCCAC	AGTCAGTCAG	CC	GGACTACHVGGGTWTCTAAT
Boxo3C.86	806rcbc86	CAAGCAGAAGACGGCATACGAGAT	TCCCAGAACAAC	AGTCAGTCAG	CC	GGACTACHVGGGTWTCTAAT
Boxo4.49	806rcbc49	CAAGCAGAAGACGGCATACGAGAT	TAGGAACTGGCC	AGTCAGTCAG	CC	GGACTACHVGGGTWTCTAAT
Boxo5.37	806rcbc37	CAAGCAGAAGACGGCATACGAGAT	TCGAGGACTGCA	AGTCAGTCAG	CC	GGACTACHVGGGTWTCTAAT
Boxo5C.74	806rcbc74	CAAGCAGAAGACGGCATACGAGAT	CAAGCATGCCTA	AGTCAGTCAG	CC	GGACTACHVGGGTWTCTAAT
Boxo6.25	806rcbc25	CAAGCAGAAGACGGCATACGAGAT	TAGGATTGCTCG	AGTCAGTCAG	CC	GGACTACHVGGGTWTCTAAT
Boxo6C.62	806rcbc62	CAAGCAGAAGACGGCATACGAGAT	GAATCTTCGAGC	AGTCAGTCAG	CC	GGACTACHVGGGTWTCTAAT
