# Supplementary material for: Enrichment of Verrucomicrobia, Actinobacteria and Burkholderiales drives selection of bacterial community from soil by maize roots in a traditional milpa agroecosystem
Source: PLoS One. 2018 Dec 20;13(12):e0208852. doi: 10.1371/journal.pone.0208852 (PMC6301694; doi:10.1371/journal.pone.0208852)
Supplement: S2 Table — (PDF) [file pone.0208852.s002.pdf]

S2 Table. Sequences per sample

Sample	raw	filtered	denoised	merged	non-chimeric
M1	96442	82180	82180	50857	47987
M2	86123	73854	73854	44344	41471
M3	91042	81773	81773	54282	49545
M5	88699	77052	77052	46581	44017
M6	75986	66134	66134	36876	34870
C1	101274	86968	86968	54916	51168
C2	112977	98401	98401	61543	57323
C3	196560	170569	170569	117763	104479
C5	113402	96912	96912	64240	58579
C6	122541	106589	106589	70644	65306
Total	1085046	940432	940432	602046	554745

Note: raw reads were processed using dada2. Samples starting with M were collected at the roots of maize plants and samples starting with C were collected from bulk soil, collected at 30 cm from each plant.
