# Supplementary material for: Enrichment of Verrucomicrobia, Actinobacteria and Burkholderiales drives selection of bacterial community from soil by maize roots in a traditional milpa agroecosystem
Source: PLoS One. 2018 Dec 20;13(12):e0208852. doi: 10.1371/journal.pone.0208852 (PMC6301694; doi:10.1371/journal.pone.0208852)
Supplement: S3 Table — (PDF) [file pone.0208852.s003.pdf]

S3 Table. Length distribution of the clean, pre-pocessed, quimera-free DNA Sequence Variants

Length (nt)	# of Secuence Variants
238	4
245	2
246	1
247	9
248	215
249	3593
250	22
251	2
282	1
Total	3849
