# Supplementary material for: Enrichment of Verrucomicrobia, Actinobacteria and Burkholderiales drives selection of bacterial community from soil by maize roots in a traditional milpa agroecosystem
Source: PLoS One. 2018 Dec 20;13(12):e0208852. doi: 10.1371/journal.pone.0208852 (PMC6301694; doi:10.1371/journal.pone.0208852)
Supplement: S4 Table — (PDF) [file pone.0208852.s004.pdf]

S4 Table. Soil analysis parameters.

Parameter	Value
Sand	29%
Clay	39%
Silt	32%
pH	6.7
Organic matter	3.61%
N-NO3	47.4 ppm
P-PO4	196.6 ppm
K	289 ppm
Ca	7680 ppm
Mg	570 ppm
S	20 ppm
Fe	8.8 ppm
Cu	2.2 ppm
Zn	11 ppm
Mn	5.5 ppm
Na	92 ppm
