# Supplementary material for: Enrichment of Verrucomicrobia, Actinobacteria and Burkholderiales drives selection of bacterial community from soil by maize roots in a traditional milpa agroecosystem
Source: PLoS One. 2018 Dec 20;13(12):e0208852. doi: 10.1371/journal.pone.0208852 (PMC6301694; doi:10.1371/journal.pone.0208852)
Supplement: S5 Table — (PDF) [file pone.0208852.s005.pdf]

S5 Table. Wilcoxon tests for the differences in abundances of phyla (Only the 14 most abundant phyla were tested.

Phylum	Control	Maize	P-value
Proteobacteria	0.3607±0.0190	0.3730±0.0125	0.421
Acidobacteria	0.2324±0.0101	0.2057±0.0591	0.222
Actinobacteria	0.1366±0.0324	0.1392±0.0724	0.548
Bacteroidetes	0.0474±0.0084	0.0601±0.0105	0.055
Chloroflexi	0.0524±0.0004	0.0401±0.0048	0.095
Planctomycetes	0.0470±0.0025	0.0438±0.0146	0.151
Verrucomicrobia	0.0266±0.0057	0.0367±0.0095	**0.008**
Gemmatimonadetes	0.0359±0.0042	0.0240±0.0128	**0.032**
Nitrospirae	0.0112±0.0033	0.0065±0.0040	0.095
Cyanobacteria	0.0078±0.0099	0.0086±0.0082	0.548
Thaumarchaeota	0.0079±0.0021	0.0082±0.0035	1.000
Firmicutes	0.0047±0.0019	0.0030±0.0019	0.310
WS3	0.0036±0.0041	0.0027±0.0005	0.548
Armatimonadetes	0.0032±0.0012	0.0037±0.0004	0.421

Note: Values are medians ± inter quartile ranges. Highlighted p-values are considered significant at the 95% confidence level.
