# Supplementary material for: Enrichment of Verrucomicrobia, Actinobacteria and Burkholderiales drives selection of bacterial community from soil by maize roots in a traditional milpa agroecosystem
Source: PLoS One. 2018 Dec 20;13(12):e0208852. doi: 10.1371/journal.pone.0208852 (PMC6301694; doi:10.1371/journal.pone.0208852)
Supplement: S6 Table — (PDF) [file pone.0208852.s006.pdf]

S6 Table. Wilcoxon tests for differences of Sequence Variants (SV) between Maize-root samples and Controls.

SV	Taxonomy*	Maize	Control	pvalue
b3f31ece386a0592859eb3d102987f9f	Family Nitrosomonadaceae	0±0	90±16	0.0158730159
32bc11b0aa283e3d17c6636c76ad4b74	Class Acidobacteria	0±0	82±16	0.0079365079
e0fc752ac14b8fc73b082430ddd5c660	Class Betaproteobacteria	0±0	62±14	0.0158730159
a544411239b3db5d010ee4c445be0df1	Class Acidobacteria	0±0	51±7	0.0079365079
e2d0f07a3a468929944b01f9ef0a3a82	Class Acidobacteria	0±0	47±17	0.0079365079
d0c86903b8fd0352196d1975ae371e41	Order Rhizobiales	0±0	46±22	0.0079365079
435407f65258b5c108bb578a11eedfd4	Family Gemmatimonadaceae	0±36	45±8	0.0079365079
cdc407c13a646e74e35fb1fac4ddf75e	Order Xanthomonadales	0±20	45±6	0.0158730159
f219e981cc92ace9a87ec52bb6aed3c8	Genus Devosia	0±0	42±14	0.0317460317
da9e3abfe1dfec7f41111d733754c0c6	Order Rhizobiales	0±0	39±7	0.0079365079
d128e023534f57584bb2b819de9f6a5b	Phylum Planctomycetes	0±0	38±11	0.0317460317
501d4c06a8a698f0f6e2b9ba720563b5	Class Acidobacteria	0±0	37±7	0.0079365079
05a683b4d7a634a24215077043da06eb	Class Acidobacteria	0±20	36±8	0.0158730159
5aaf22ed43ac6780cda73c6dc35ba24e	Family Nitrosomonadaceae	0±0	36±13	0.0317460317
8643fc7a49aa927e2f48e7db617671bd	Class Acidobacteria	0±0	34±6	0.0079365079
e9bbd9edb7df0df7197d8de766843a3d	Family Nitrosomonadaceae	0±0	32±4	0.0079365079
3995a3d33c949a95fffd41e9df3d1e66	Family Gemmatimonadaceae	28±32	57±17	0.0158730159
bf6fea04809a5226b3d19e6586913864	Family Sandaracinaceae	0±0	27±16	0.0079365079
969911a14519cecd682f0db9a4167350	Family Polyangiaceae	0±0	26±6	0.0079365079
34a71e8fff0373ae73ecc59ff4e88e2d	Family Planctomycetaceae	0±0	26±2	0.0158730159
3838798dcf326c3ce89c188fa8b0ca17	Class Elusimicrobia	0±0	25±8	0.0079365079
992de1dd3b965829ab8d75f3ced96eaa	Class Deltaproteobacteria	0±0	22±18	0.0079365079
4a6e78fa80a4388d9b1f8a9b286cee42	Order Xanthomonadales	41±14	63±7	0.0317460317
2c8b3833fcc16d7321a365244e39db9f	Order Rickettsiales	0±0	21±0	0.0158730159
70a34e043a7d156d7d304f5cb0ca4c84	Order Rhizobiales	28±1	46±10	0.0317460317
ca354988523e3c4bfbacfe85b770dbb9	Phylum Verrucomicrobia	0±0	16±6	0.0079365079
fee58eab035a352e82a65cc489d07777	Genus Hirschia	0±0	16±6	0.0158730159
2e8f0513d1d916c4a37bc57cd2fb9b18	Genus Opitutus	28±7	15±9	0.0158730159
d9448a1b73de1f09ec6868ac89da55f9	Genus Dongia	41±6	24±8	0.0079365079
655ed3034655eb6b0c7c467d3570422d	Class Phycisphaerae	61±13	44±13	0.0317460317
1a60ebdb9ea705580ed21baccdb83707	Order Sphingomonadales	90±28	63±16	0.0079365079
96325fa080ec907df171349fe5c3b90b	Order Solirubrobacterales	81±8	53±5	0.0079365079
639792d7a10a2fbff57393b59e3abcaf	Genus Flavitalea	59±19	30±7	0.0079365079
f965016a2233488c2e69348bbb472b57	Order Xanthomonadales	30±9	0±13	0.0158730159
b961b6b8c7d19b4ec13d2653e8d83689	Genus Caenimonas	59±8	28±9	0.0317460317
d929041c3e2976504a9a15ee3122cebe	Family Comamonadaceae	72±11	34±7	0.0079365079

20a595eae782cb9fff2847b195e1d97f	Genus Patulibacter	66±19	28±37	0.0317460317
5b14dbf098678cd784592d7c29237332	Family Comamonadaceae	94±66	55±16	0.0079365079
421dfc8e26b7707e0156ca09409c6571	Genus Ramlibacter	91±10	51±18	0.0079365079
d983c3737f8e5e7cb2c569592a4fdca3	Genus Chthoniobacter	59±29	17±7	0.0079365079
6523cdd46aa2eb9657437c3a80888a88	Genus Solirubrobacter	159±52	109±22	0.0317460317
9e734cdb69272015147b754d14446964	Family Intrasporangiaceae	78±44	26±27	0.0158730159
6ede21b8c056dcf441c136a0f5f48fb5	Genus Blastococcus	57±1	0±27	0.0158730159
702853d835836536115f097235fe6693	Genus Microvirga	100±49	41±20	0.0317460317
3e2895c199f191ef63b524f1d9e125d6	Genus Rhizobacter	122±69	62±21	0.0158730159
80841afd936d44406d50ce6429de0705	Family Comamonadaceae	63±40	0±0	0.0079365079
50ea59e716ce9e33f2050caba48a6619	Family Comamonadaceae	70±7	0±15	0.0079365079
518b2949de955b8965d108427323cee9	Genus Ramlibacter	118±12	45±4	0.0079365079
714bf623d76c84f057dfbb70eaac6056	Family Verrucomicrobiaceae	74±2	0±32	0.0317460317
dcae6315654c1bdc94ec08bf335c57dd	Genus Chthoniobacter	75±39	0±37	0.0079365079
bc59be3577d33ed21e0ea52d4fbf4836	Order Burkholderiales	111±16	35±5	0.0079365079
ef5de1a9261c8e157f0f8692342f7971	Family Comamonadaceae	78±33	0±27	0.0317460317
8d642882b72ed3482ff3c05c3cd203f9	Genus Chthoniobacter	128±20	47±14	0.0079365079
1989bb0c2d1ac1cf8991b40c25392037	Genus Arenimonas	89±14	0±64	0.0079365079

Note: Values are medians ± interquatrile ranges, from a Sequence Variant table rarefied to equal read numbers between samples. Tests with p-values < 0.05 are shown. *The lowest taxonomic affiliation available is shown.
