# Supplementary figures and images for: Enrichment of Verrucomicrobia, Actinobacteria and Burkholderiales drives selection of bacterial community from soil by maize roots in a traditional milpa agroecosystem
Source: PLoS One. 2018 Dec 20;13(12):e0208852. doi: 10.1371/journal.pone.0208852 (PMC6301694; doi:10.1371/journal.pone.0208852)

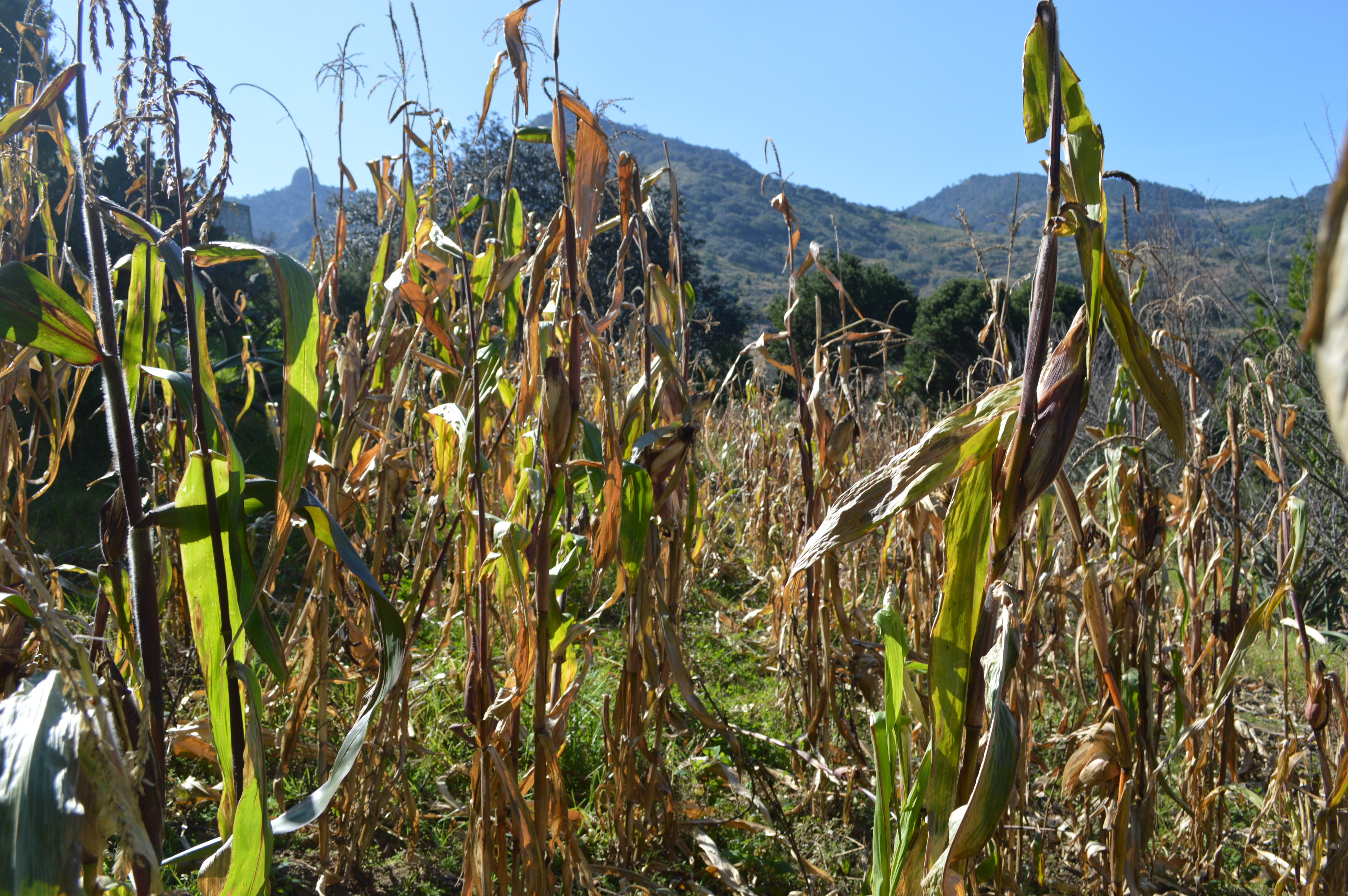

Supplement: S1 Fig — Image of the milpa. (JPG) [file pone.0208852.s007.JPG]

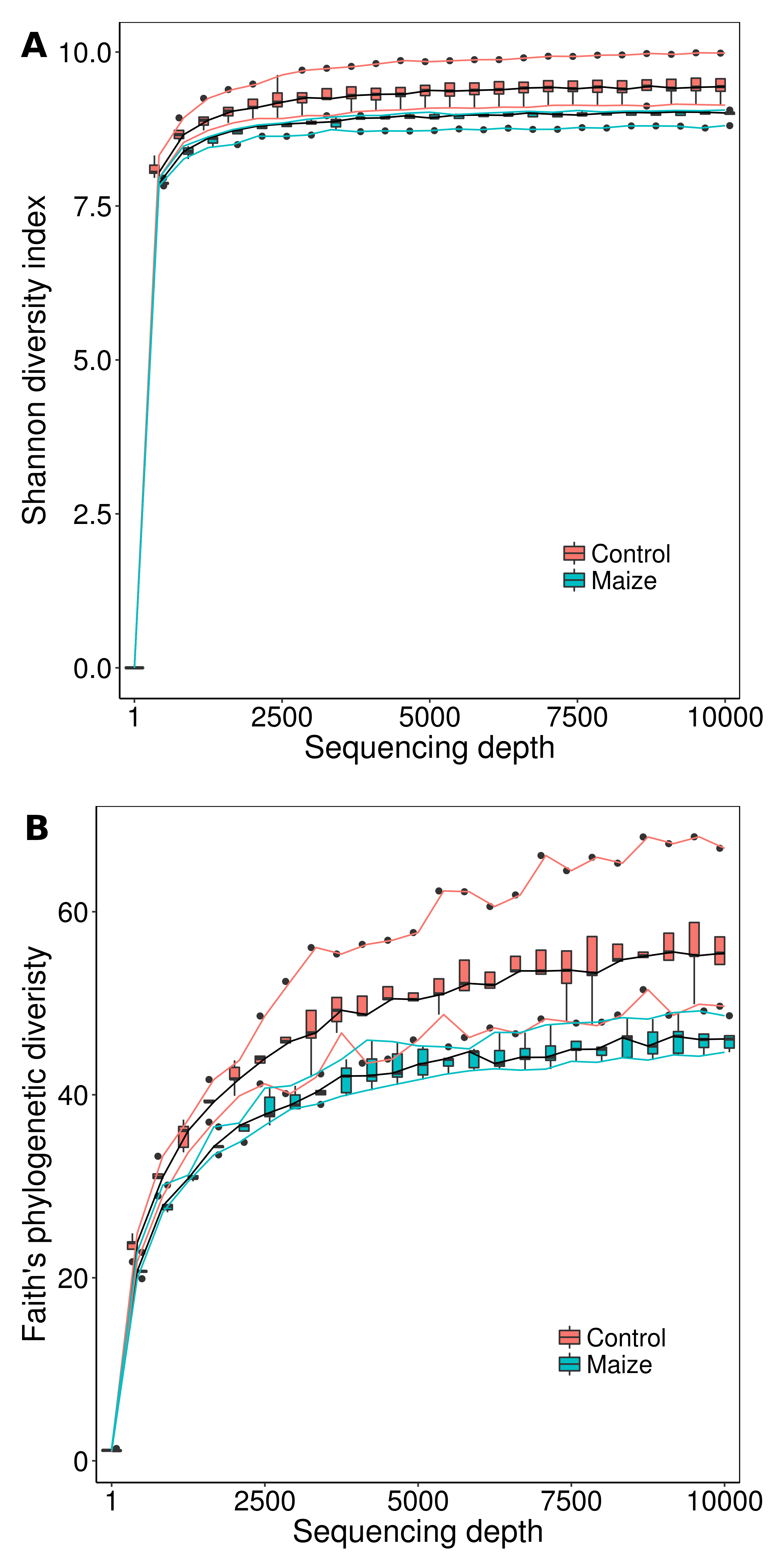

Supplement: S3 Fig — Rarefaction curve for A) Shannon diversity index and B) Faith’s phylogenetic diversity index. At each sampling depth 10 random samples were taken, and the indexes for all 5 samples per group are shown as boxplots (50 values per box plot). Maize-root soil samples are shown in blue, and controls in red. Continuous lines show the most extreme values at each sampling depth. (TIFF) [file pone.0208852.s009.tiff]

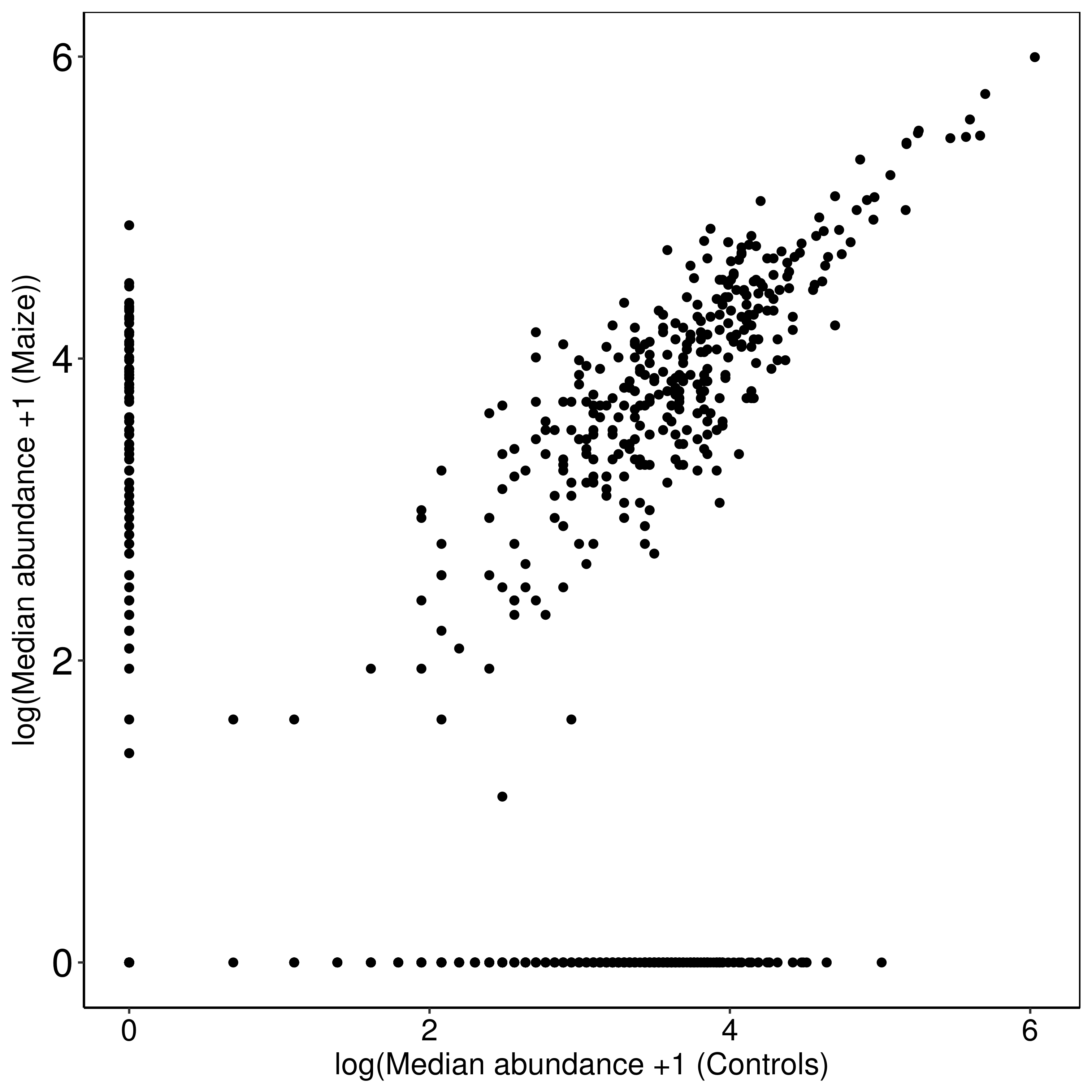

Supplement: S4 Fig — (TIFF) [file pone.0208852.s010.tiff]
