# Supplementary material for: Enrichment of Verrucomicrobia, Actinobacteria and Burkholderiales drives selection of bacterial community from soil by maize roots in a traditional milpa agroecosystem
Source: PLoS One. 2018 Dec 20;13(12):e0208852. doi: 10.1371/journal.pone.0208852 (PMC6301694; doi:10.1371/journal.pone.0208852)
Supplement: S1 Interview — (PDF) [file pone.0208852.s011.pdf]

S1 Interview Transcript. Verbal permission to publish this interview was granted by all participants.

(This part of the interview was done in the area where the study was performed)

Ricardo Garibay (RG): What is your name?
Pedro Rivera (PR): Pedro, Pedro
RG: Pedro Rivera
RG: You tell me you plant two kinds of native maizes, or how many?
PR: They are three[1]
RG: Do those maices have names? Which?
PR: We only call this one Variegated (Maíz Pinto), large grain variegated, it has a late cycle
RG: How many months?
PR: They are planted in Ju… in May, it depends on the rain season, but this one is almost from May to November, December.
RG: Late cycle
PR: Late cycle
RG: And the other one?
PR: All the same, almost all have a late cycle
RG: And then, how do you call the others here?
PR: Well, I call, they call the other Coal Maize (Maíz Carbonero)
RG: Coal
PR: It is a blackish maize, coal, the other is a white maize, it is like this one but it is white, it doesn't have purple seeds or of different colors.
RG: And the three have the same cycle?
PR: Yes, they have a late cycle
RG: Then, why do they have differences between each other? What is the difference?
PR: Well, the… no… well, the difference is their color
RG: Only their color?
PR: Their color
RG: The taste, or to prepare different things, don't you use it?
PR: Well yes, that one like blackish we use it to make Atole[2].
RG: Atole, exactly
PR: We make atole, we make…, They use a lot… the white one is used to make pozole, the other one is almost for atole and this one is for maize, for tortilla
RG: And, what other products do you plant? I am seeing that you plant there…
PR: We plant this been
RG: What kind of bean is it?
PR: This one is called bean. But, I don't know which variety it is. Entangled bean.
RG: You tell me this soil has a lot of clay
PR: It has clay.[3]
RG: And what other soil-type is there here at the community?
PR: Well, this one, and the other is like sandy, like grayish, and in a moment, we will go see it.
RG: Hey, Don Pedro, I saw you also have fava bean
PR: Yes, I also plant fava bean
RG: And let's see, walking through the land I saw you also have fruit trees here. What are they?

1At the harvest time, we actually found four kinds of maize.
2Prehispanic hot breverage, non-alcoholic, prepared with maize flour and water and unrefined cane sugar, and can contain fruits or other ingredients.
3This is the area where the samples were collected.

PR: I have here this plum tree, I have this, this pear, I have apples and bitter-berry
RG: All right, what else, what else?
PR: Tejocote and chabacano
RG: Look, and only that, and then to delimit the, the, the parcels, there is maguey.
PR: Yes, this maguey. We use maguey to stop erosion, but yes, to avoid that the soil erodes.
RG: Exactly, OK
PR: We put stone barriers, and then we put maguey, and we plant fruit-trees and that helps us to stop erosion and also avoids that the wind passes strongly, they are like wind-breaking barriers
RG: Wind-breaking barriers
PR: Aha
RG: And your land is like in terraces
PR: Yes
RG: Only three terraces
PR: Yes, there are three terraces
RG: And you built them along the years
PR: Yes, along the years, yes
RG: Because the soil… and why is the soil so nearby so different, here and there?[4]
PR: I think it is… because… because well yes, here the detail I have seen, is like… by zones… from here to the hill where I live it is this kind of dirt.
RG: Which one is better?
PR: Well look, it is what we are seeing, many people like this kind of soil, it is like sandy, with only one rain the soil gets well humid, and that one takes longer, but the detail is that this one, well, I think it is better, because when it rains, even though it takes longer, once it is humid, it is difficult for humidity to escape, it can last two or three months without rain, this one then starts to dry, like getting wrinkles.
RG: The sandy one gets dry.
PR: Yes, aha, and this other one takes like two or three months, and it is like nothing
RG: And how do you call the other one?
PR: Well, we call it clay, or red soil
RG: The red clay takes long to dry, it retains humidity better
PR: Exactly, but this one when we plant, this is an advantage that with a strong rain, it filters rapidly down below, and this one not, the first one starts like the water to drain, the water drains a lot.
RG: Hey, Don Pedro, and about extension, how large are your parcels?
PR: That I plant
RG: Aha
PR: Like, like 4 hectares, I thing like 5, but here I have some, some there, some down there and others in a different place, that they call The Farm (El Rancho) over there
RG: Like that people have their parcels in different places?
PR: Yes, yes, they have different lands
RG: Why are they small land pieces?
PR: They are private properties, small parcels
RG: And they are in a slope?
PR: And yes, there are places where it is, like slopes, there are places like flat, for example, there where I was over there, I have a cousin that has some lands like flat and the soil is like this one, like sandy soil
RG: Yes, and the production you have in these 4 hectares are enough for your consumption?
PR: Yes
RG: Of your family

4They are talking about another milpa, that Don Pedro has at a distance of about 200 m.

PR: Yes, what helps us is being in a tall place, here there is rain almost the whole year, but it is already going to El Santuario, I do not know if you have seen, by the topography of the hoops, when the cloud comes, all the cloud and the precipitation here stays, the clouds are already passing by, for that side there, for the valley, but the empty clouds are passing by, without everything, everything rushes here, and on this side, everything passes without humidity, with nothing.
RG: This location suits you
PR: Yes, for the topography of the land, is full of hills here and here.
RG: Yes
PR: and this side Veracruz is located, all the humidity that comes from Veracruz here arrives, and already for that side, I do not know if you have seen over there and it is desert and we are very close.
RG: Yes
PR: Yes, from here to Cardonal it will be like half an hour.
RG: We are surprised by the sudden change.
PR: and there are some people for example who come to leave building material, they arrive here wearing t-shirt, where it's very cold and it's raining, and they say –it's very strange because it's not raining there, if we see that there is cloudy but the clouds are up and not here.
RG: Another one, so it stays recorded: what is the name of this community?
PR: Here it is called El Boxo, Cardonal municipality.
RG: And is the population Hñähñú[5]?
PR: It is a Hñähñú population, we speak Hñähñú
RG: That is good, is there a bilingual school here?
PR: Yes, here it is, up here there is a bilingual school
RG: And does it operate bilingually?
PR: Yes
RG: Are there bilingual teachers?
PR: Yes, there are bilingual teachers, they speak Hñähñú and Spanish.
PR: Almost the plants not get sick.
RG: And when it sick, of what?
PR: For example, the more plant susceptible to diseases is the broad beans. It gets sick when it does not rain, I do not know what is the plague but the plant develops a gray powder.
GR: It´s like a fungus.
PR: Exactly, look here, here is this!
RG: Perfect!
PR: And when it rains a lot and the plant have a lot of humidity, the broad beans get sick but it is black. The maize almost never gets sick, it is very resistant. The only thing that affects us a little are the birds, when we start to harvest, they also want to eat. The worms, there are little worms that come in here, but it also helps us a lot, because we put them in little bottles.
RG: Yes
PR: We roast them in the comal and we eat them.
RG: What are these worms called? Do you know?
PR: They call it a corn worm, but I do not know its scientific name.
RG: In Hñähñú,
PR: This one… they say chola in dialect ... it's like little worm
RG: Ok
PR: Raise it up!
RG: Then, it is edible
PR: Yes, it has a lot of protein. We can prepare sauces with those worms.

5Hñähñú is the name Otomi people use for themselves

RG: Of course
PR: Or simply, the worms are roasted in the comal and we eat them raw
RG: You take advantage of some of the herbs too
PR: Yes, also the quelite is cultivated here.
RG: How many types of quelite are there in this parcel?
PR: Here we can find up to four types.
RG: Four
PR: All these are edible
RG: and mushrooms?
PR Mushrooms, but, here the mushroom of the maize, but there are also mushrooms in the hill
RG: Yes, of those
PR: Yes
RG: But not huitlacoche
PR: Yes, but….
RG: For example, in this mazorca, what is this black?
PR: This black is like a mushroom
RG: mushroom?
PR: Yes, but this has been eaten by a bird!
RG: Yes, but this black is a mushroom
PR: Yes, is a mushroom, for the humidity
RG: Does this fungus affect them in agricultural production?
PR: No
RG: No
PR: No, this mushroom developed due to humidity
RG: Yes, the plant is already decaying
PR: Look here in the back
RG: Yes
PR: Yes, but this has been chopped the bird.
RG: Yes, there are some insect inside
PR: Yes, but that is normal. Since the beginning of the harvest the bird must eat 5%
RG: Besides the bird, is there some other animal that attacks at the corn?
PR: Well, also the dogs
RG: Dogs
PR: Yes, dogs, dogs also like the corn a lot, because it is sweet
RG: Corn is bitten by the dogs
PR: Yes, also the badger, there is an animal that is called a badger. The badger is like a raccoon.
RG: Yes
PR: Badger also eats maize. But not here. In places where we have milpa on the hill, there it is, but here almost no.
RG: Yes
PR: The bird eats it. Then the corn comes out like a little mushroom because of the excess moisture
RG: There are some one in the village that buy to your maize? For consumption
PR: Well…
RG: Or, just have it?
PR: Well, there are people who have maize left over and they sell it. So, that means yes but between neighbors or people that coming from the city. People who want to eat organic foods. Yes, we also used dung to fertilize the soil.
RG: Sure
RG: This is the huitlacoche

PR: Is the huitlacoche, yesterday we ate it
RG: When the huitlacoche grows?
PR: In this season, when for example is raining and that the mazorca is present and when it starts to rain.
RG: The huitlacoche
PR: But, there are years. This huitlacoche goes out a lot when there are good years… that it rains a lot.
RG: Yes
PR: When it does not rain so much, the huitlacoche does not come out
RG: And, you recover it?
PR: Which one? the huitlacoche
RG: Yes
PR: Oh yeah, we can even dry it.
RG: It is like consecrated, right?
PR: Yes
RG: Because it is scarce
PR: Yes, and it is very expensive!
RG: Yes, enough and you can dehydrate and then hydrate it
PR: In January, February when there are not huitlacoche, we added water and we prepared it to eat.
RG: It is customary to dehydrate
PR: Yes, we also hang it!
RG: strung up
PR: Yes, the huitlacoche is dried. It is sells for 40 or 45 pesos per kilo approximately in Mesquilpan, Lately, there are many people who are worrying about their diet.
RG: Yes
PR: People who are already preferring to buy organic foods, natural, that does not have fertilizer or herbicides.
RG: Do you apply any chemical to the soil?
PR: No
RG: Nobody from here?
PR: We fertilize the soil with dung of sheep, bulls, goats… but mainly with sheep, most people have sheep here.
RG: Yes
PR: And for example, for weed control we used instruments like shovel, azadon… we don't used herbicides.
RG: Well yes, it is organic.
PR: Yes, is organic.
PR: And bean and broad beans are crops associated with maize?
PR: Yes, we put those plants here. Here the chapulin does not arrive yet. In Santuario, another small town that is more ahead, lately the chapulin has arrived already, but to our town not (El Boxo), because the chapulin does not like the climate of here.
RG: Ah!
PR: As it is very cold here.
RG: Ok
PR: Recently, some biologists came here, I think Dutch, they were asking me how could they control that plague. I say, we do not know how, but take some chickens!
RG: Some chickens, you are right!
PR: Yes, they are like biological control!
RG: Yes.

PR: Here, the climate is very cold and it is very high, and I think that this type of climates is not good for chapulines.
RG: Yes, they like the desert?
PR: Yes, in the desert, a hot place.
RG: A place with humid weather?
PR: Yes.
RG: That tuna is edible?
PR: Yes
RG: Is not the xoconostle that?
PR: No, here the xoconostle is not cultivated, this tuna is from a hill field, native to this region.
RG: Yes, but there are also several varieties.
PR: Of tuna?
RG: Tuna.
PR: Yes, there are different, there is another thicker one that is used for animal feed, this can also be used.
RG: The tuna or the penca?
PR: The penca, the tuna is eating bye the people and the birds.
RG: Yes.
PR: Here, the tuna is produced little, that is, we plant prickly pear for tuna, but the quality of it is not good, it is not very good, it is not very sweet.
RG: Ok
PR: And the one of Cardonal is better, as there is not much rain this very concentrated.
RG: Concentrated.
PR: Here we recently produced a lot of pear.
RG: Pear.
PR: This is the pear, the pear is usually produced a lot here, as well as the plum, apple, tejocote.

RG: What do you think has helped you live this long?
PR: There are still people that here, there are people that have managed to lived up to 90 to 100 years, even more. There is a person that lives here, must be 100 years old o even more than that. His grandmother is still alive. These people don’t like the processed meat, they do not eat sausages, do not eat ham, their food is this, the “mushrooms” like you name it, the squash, when they’re ripe and huitlacoche.
RG: How many kinds of mushrooms do you recall are still wildtype?
PR: Edible mushrooms?
RG: Yes
PR: 15
RG: 15 types of edible mushrooms.
RG: How many types of quelites do you recall there are?
PR: (He speaks in his native language) like 5 types of quelites
RG: Do you recall harvesting amaranth? Or people before you?
PR: No, people used to harvest Green tomatoes, they still do, what happens is that we haven’t planted tomatoes in this land, but sometimes from what we buy there is also a tomato that we only dry, take its seeds and it also grows (the tomato). But this was done by other people, we don’t do that anymore, we are from another generation.
O: It strikes me odd that you didn’t plant squash.
PR: I also plant squash, of the kind that they call “chinan”, well, we have down there, if you want we can take a walk there so you can see. I’ll show you there the other kind of corn that I’m telling you that I plant, they call it “carbonero” not “Ramon”, it’s a white with black corn, like *pinto* (spotty).

O: Pinto, carbonero. They use that a lot for atoles.
RG: For atoles the pinto, carbonero. But also for tortillas. Here in this side of Actopan I've seen a billboard that says that they sell "denim" tortillas. Denim tortillas (and I went to see how those tortillas look like made with that carbonero corn.
RG: Denim tortilla?
PR: When you grind that corn, the tortilla gets the color of denim pants.
RG: Oh blue, hehehe (unintelligible voices in the background, people walking and chatting in the background apparently in their native language)
PR: Watch it, watch it, be careful, be careful, hold, right here, be careful.

(They move to another location, to the other *milpa* that is at about 200 m distance)
GR: These were cut, then?
PR: Yes, its all plain here now. As I tell you, it rained a lot this year and it was windy as well, because its too tall, it collapses.
Woman: And that one, the pumpkin is used for food, yeah.
PR: Yes, to prepare as a candy.
Woman: Yes with sugar.
PR: Yes, it was used for example, in the last day of the Death, we prepared pumpkin candy.
Woman: Its prepared with caramelized sugar.
PR: Yes. Here we also plant fruit trees. Last year. But we have fruit trees here too, look, we plant them as a wall, with maguey, and then we intercalate trees.
GR: So its a lot of work, first build the terraces for the walls.
PR: Yes.
GR: To hold the soil.
PR: Yes, but it helps a lot, because that way the soil is not eroded and gets kind of plain.
GR: Sure.
Woman: That milpa over there is greener.
PR: Yes, that one has more manure, manure makes everything better, last year we used it here. We had bulls and we fed them here, kept them here and here stayed their feces.
GR: Don Pedro, are these all private lands?
PR: Yes.
GR: The public lands are over there?
PR: Yes, those start after that canyon.
GR: What about communal lands?
PR: There is only one, but it has plenty of water, it is not big, maybe 3 or 4 hectares, and the village has 6 hectares.
GR: That belongs to the village?
PR: Yes.
GR: Those lands have free access?
PR: There is a church, a water basin, and a community sports field.
GR: Oh I see.
PR: Yes, we have 6 Hectares i think, most of the village is private and then there is a big land that belongs to one farmer, that is more than 70 hectares.
Woman: A different huitlacoche
PR: Yes it is a different huitlacoche and it is bigger.
Woman: Because of the humidity.
PR: Yes, that is a different variety.
GR: It has a beautiful color.
Woman: Do you want to hold it?

PR: You can cut it if you want, take it as a gift, maybe you can use it as a sample, take it, take the whole plant.
Man: How do you say 'good afternoon' in Otomi.
PR: *Ide*
Man: *Ide*
PR: And 'good morning', *tiajil*
Woman: *tiaj*
PR: *Tiajil*, good morning
GR: But take it, take a picture with it.
PR: Yes, this is another seed we have.
GR: For storing.
Woman: Here is one with more colors (pinto).
PR: Yes, its colorful, sometimes with the wind…
Woman: Aha.
PR: Like this one, up here, and that one down there, I planted purple corn, I mean… Carbonero maize as we call it, and then we planted Pinto, and sometimes they contaminate with pollen.
GR: Aha.
PR: Or with worms, they carry the pollen, they cross and it comes out like that. Half white with Carbonero and it results like that.
GR: To avoid crossing, you would have to wait 15 days between planting one and the other.
PR: Aha
GR: So when one releases pollen, the other is not contaminated.
PR: Aha
GR: That is what they do in other places.
PR: And that corn is very tasty.
Woman: The mixed one.
PR: No, both of these, or the white one but, you can't compare one from here with the ones irrigated at Mesquilpan.
GR: Of course.
PR: The ones from Mesquilpan are bigger, big like that, but is not similar to this one.
GR: The flavor?
Woman: Yes.
PR: We find that the ones from Mesquilpan tasteless, not this one. With those you have to add mayonaise, chilli, cheese, for it to be acceptable and this one you can it alone, it is sweet, you can eat 4 or 5.
Woman: And I bet it is very nutritious.
PR: Yes
Woman: The difference.
PR: It has to be.
GR: No one here buys corn from Conasupo, the guvernmental store?
PR: The reason is we use it mostly to eat ourselves, and there is no left, just a couple producers have some left and they sell it to neighbors.
GR: Yes, only to neighbors?
PR: And some people that come from Mexico City, form Pachuca or Queretaro.
GR: But you sell corn.
PR: Yes, but the corn I sell is from Queretaro.
GR: From Queretaro? That is rare, because you have producers closer, at Mezquital Valley. Why don't you bring it from there?

PR: That is due to the water they use. The one from Queretaro is underground water. And at Misquilpan the water they use is from…
Man: What do they call this kind of water system at Queretaro?
PR: That is irrigation with sprinklers.
GR: Its drip irrigation, and here is from channels?
PR: Here the irrigation is from the river, but the Tula river is very contaminated. If you look at the seed, it has dark spots in the front, where it attaches to the corn, and the people notice.
GR: And people here does not want to consume corn from Mezquital Valley.
PR: No, because they use contaminated water, from the river, that river comes from Mexico City.
GR: And that corn is the one we buy at Mexico City.
PR: Also, the people here look at the corn and they know it is from Queretaro, and the water is from underground reservoirs.
GR: Of course.
PR: And the water is clean.
Man: You live in Mexico City?
RG: I do.
Woman: You eat those vegetables and that squash.
PR: He lives in Mexico City. In what area?
GR: Tlalpan
PR: Near the Mexico-Cuernavaca highway.
PR: What is your brother's occupation?
MAN: He worked for the army, but he retired and he is with the federal police now.
When he comes, we cook a chicken, we like to buy the chickens alive, and then we kill them, peel them an when he comes he eats with us, and he thinks it is the best food.
GR: jaja
Man: He says in Mexico City chickens are colorless and flavorless, but here it is excellent.
GR: Of course.
Man: It is the same with everything.
Woman: Everything.
GR: Don Pedro, I have one doubt. The community plants their own local maize because they like it, maybe for atole, or for tortilla, but their milpas are not enough for their requirements, and so they buy corn from you.
PR: Exactly.
GR: So they need more corn.
PR: The thing is, I sell my product to a tortilla store in the city limits, and many people recently feels its not profitable to plant corn, they feel is better not to invest, most of the time they don't lose money but they don't win either, but it is a lot of work invested, so people prefer to have other occupations, like in construction, which is more profitable.
Man: And they just buy the corn.
PR: Exactly.
GR: But there are enough laborers here in the community and you could pay for the job.
PR: Yes.
GR: Yes, not many people migrate to the US?
PR: Sometimes the ones living in the US, send money to someone to work in their lands.
GR: And they do that with animals, no tractors?
PR: Yes, with animals.
GR: Mules.
PR: Some of them use tractors, but just a few, because most of the milpas here are tilted, in the hills, but in the ones, that are plain they use tractors. However, we have noticed that when we use tractors too

much, we modify the structure of the soil, it wears away. One time my neighbor used tractor in his lands, and last year his soil was like powder, useless and this year he planted corn, and he got no production.
Man: That is a shame.
PR: No production, because he brought technology, and tractors really really affect the soil, it may even kill the microorganisms, i don't know.
GR: And the worm?
PR: And this year he got no production.
Man: Maybe the movement of the tractor affects the very few nutrients in the soil from the manure, and spoils it.
PR: So we only use animals, horses.
GR: And you have separated milpas depending on the corn variety you want to plant?
PR: Yes
GR: So they don't cross.
PR: Yes, so they don't mix. Here i I have only this one color, and up there I have a different kind.
GR: What is the name of this corn again?
PR: This is carbonero.
GR: And what it its name in the Hñähñú languaje?
PR: It is *Petel*, it is like black corn in spanish. We also have the pinto and the white.
GR: And those are the three races here?
PR: Some farmers plant smaller seeds, its white but smaller, and it has a shorter cycle. That one, for example, is planted in May and it is ready by September. It is a short cycle, a small seed and a tiny cob. That one is used in Santuario, because they have a different weather, they have to take advantage of their rain seasons. It rains less there, so they need a short period maize. Here we use the large seed corn because it rains all year.
GR: And that is just one cycle per year?
PR: Yes. And if it rains like now, many people plant oats when they finish harvesting corn.
Woman: Oh.
PR: Yes, oats.
Man: So they can earn a little bit more.
PR: It has been 6 or 7 years with rains all year, so there has been chance to introduce oats. It used to rain less, and now maybe 3 or 4 years ago, it started raining like before. The canyons are full of water, they used to be dry, there was no water. In the year 2000. But here, thank God, due to the terrain and the topography of the hills, we have had plenty of water in recent years. Just before the day of the dead, it rained 2 or 3 days, day and night, and 8 days ago, so now the canyons are full of water, and that is why these corns have not dried. We are waiting for the rain to stop to finish harvesting.
GR: So you stop the harvest?
PR: Yes.
GR: You pickup everything in sacs.
PR: Yes.
GR: And then the stubble?
PR: We give that to animals, sheeps. And also pigs, bulls, horses.
GR: Is there a Saint or a party related to the harvest?
PR: Yes, the village fair.
GR: When is it?
PR: January 6th.
GR: The celebration of the 3 wise men?
PR: Yes, many people go and give thanks, they bring corn and they come from surrounding villages too.

GR: Is this a gathering center for that ceremony?
Man: Not really, but we have guests, they come and they are thankful, and they bring their corn.
GR: That is the main community festivity?
PR: Is the celebration of our main Saint. Many people, because they are grateful of the lands they received, bring beans and corn.
Man: And that is a tradition for many years?
PR: Yes, many years.
Mans: In religion we have mover forward, we built a chapel, so we concentrate the cult. We used to do it in particular homes. So you are invited on January 6th to come and have a look.
Woman: We would love to.
PR: He is from the committee. Seriously, come and check it out, many people come and bring beans.
Woman: All the varieties?
PR: You will see, a lot of people will come.
Woman: With their offerings…
PR: Exactly for the three wise men.
Woman: We will consider it.
PR: Well if you came…
Woman: We will be here on the 6th, and you can look at the ceremonies and how a lot of people come and bring their offerings, as a gratitude for their harvests, every year, every year.

GR: These guys make soil analyses, for microbes and who knows what else, then we can ask for your permission to take soil samples from your lands.
PR: Yes, as I was telling you earlier, you should have taken samples form this lands, its convenient for me, they will give me the results, if there is a lack of nutrients…
Man: Whatever we find.
PR: And every time you want to come for research, you can come to me, you have my permission to sample in my lands.
Woman: At the end of the day, the objective is to communicate our results to all of you, so you can have increases in your productions.
Man: And probably we will come back, periodically.
PR: Yes, my store is right there, where you found me, I sell construction material, I'm there all the time.
Woman: Very good.
PR: If I'm not there, you can come in anyway to my crops, you can take the samples.
Woman: Thank you.
PR: I should have told you to come this time, I want to know how good is my soil, where I grow corn.
Woman: Of course.
Man: The insects are beautiful.
Woman: Lets grab the samples, the bags.
Man: What do you think, should we sample the three of them? Up there and down here?
Woman: Yes.
PR: But, there is for example… the one with the yellow flower…
O: Chamomile
PR: Yes, but of the native
RG: Wild-type.
PR: From the wild, with those you can also make tea. For stomach ache.
O: Also, here there is *ajenco*
PR: There is also, in the hill, wild *oregano*, down here I have an oregano.
RG: Five varieties of insects also edible.

PR: Chinicuil
RG: The worm of the *maguey*
PR: Yes, worm of the maguey, also *Escamoles*.
RG: *Escamoles*.
PR: Yes, the wood worm and this one that they say the little black worm.
RG: There are already five.
PR: There's no more.
RG: Well, Don Pedro we thank you for your support. We are going to come back after because we need to make the food system of the Hñähñú community of this region.
